# The Complete Genome Sequence of *Thermoproteus tenax*: A Physiologically Versatile Member of the *Crenarchaeota*

**DOI:** 10.1371/journal.pone.0024222

**Published:** 2011-10-07

**Authors:** Bettina Siebers, Melanie Zaparty, Guenter Raddatz, Britta Tjaden, Sonja-Verena Albers, Steve D. Bell, Fabian Blombach, Arnulf Kletzin, Nikos Kyrpides, Christa Lanz, André Plagens, Markus Rampp, Andrea Rosinus, Mathias von Jan, Kira S. Makarova, Hans-Peter Klenk, Stephan C. Schuster, Reinhard Hensel

**Affiliations:** 1 Faculty of Chemistry, Biofilm Centre, Molecular Enzyme Technology and Biochemistry, University of Duisburg-Essen, Essen, Germany; 2 Institute for Molecular and Cellular Anatomy, University of Regensburg, Regensburg, Germany; 3 Max-Planck-Institute for Biological Cybernetics, Tübingen, Germany; 4 Prokaryotic RNA Biology, Max-Planck-Institute for Terrestrial Microbiology, Marburg, Germany; 5 Molecular Biology of Archaea, Max-Planck-Institute for Terrestrial Microbiology, Marburg, Germany; 6 Sir William Dunn School of Pathology, Oxford University, Oxford, United Kingdom; 7 Laboratory of Microbiology, Wageningen University, Wageningen, The Netherlands; 8 Institute of Microbiology and Genetics, Technical University Darmstadt, Darmstadt, Germany; 9 DOE Joint Genome Institute, Walnut Creek, California, United States of America; 10 Genome Centre, Max-Planck-Institute for Developmental Biology, Tuebingen, Germany; 11 Computer Centre Garching of the Max-Planck-Society (RZG), Max-Planck-Institute for Plasma Physics, München, Germany; 12 DSMZ, German Collection of Microorganisms and Cell Cultures, Braunschweig, Germany; 13 National Center for Biotechnology Information, National Institutes of Health, Bethesda, Maryland, United States of America; 14 Center for Comparative Genomics and Bioinformatics, Pennsylvania State University, University Park, Pennsylvania, United States of America; Institut de Genetique et Microbiologie, France

## Abstract

Here, we report on the complete genome sequence of the hyperthermophilic *Crenarchaeum Thermoproteus tenax* (strain Kra1, DSM 2078^T^) a type strain of the crenarchaeotal order *Thermoproteales*. Its circular 1.84-megabase genome harbors no extrachromosomal elements and 2,051 open reading frames are identified, covering 90.6% of the complete sequence, which represents a high coding density. Derived from the gene content, *T. tenax* is a representative member of the *Crenarchaeota*. The organism is strictly anaerobic and sulfur-dependent with optimal growth at 86°C and pH 5.6. One particular feature is the great metabolic versatility, which is not accompanied by a distinct increase of genome size or information density as compared to other *Crenarchaeota*. *T. tenax* is able to grow chemolithoautotrophically (CO_2_/H_2_) as well as chemoorganoheterotrophically in presence of various organic substrates. All pathways for synthesizing the 20 proteinogenic amino acids are present. In addition, two presumably complete gene sets for NADH:quinone oxidoreductase (complex I) were identified in the genome and there is evidence that either NADH or reduced ferredoxin might serve as electron donor. Beside the typical archaeal A_0_A_1_-ATP synthase, a membrane-bound pyrophosphatase is found, which might contribute to energy conservation. Surprisingly, all genes required for dissimilatory sulfate reduction are present, which is confirmed by growth experiments. Mentionable is furthermore, the presence of two proteins (ParA family ATPase, actin-like protein) that might be involved in cell division in *Thermoproteales*, where the ESCRT system is absent, and of genes involved in genetic competence (DprA, ComF) that is so far unique within *Archaea*.

## Introduction

*Thermoproteus tenax* has been the first hyperthermophilic *Archaeum* described by the pioneering work of Wolfram Zillig and Karl O. Stetter [1], [2]. The strain Kra1 was originally isolated from a solfatare in Iceland [1]. It belongs to the *Crenarchaeota* and bears important taxonomical meaning for that phylum, representing the type strain of the genus *Thermoproteus*, which is the type genus of the family *Thermoproteaceae* [1].

In addition to its hyperthermophilic lifestyle (optimal growth at 86°C and maximal growth at 96°C), the organism is able to grow chemolithoautotrophically in the presence of hydrogen and carbon dioxide [2] as well as chemoorganoheterotrophically on a variety of mono-, di- and polysaccharides, organic acids and alcohols (e.g. glucose, malate, amylase, starch or ethanol) [1]. Less efficient growth has been observed with propionate and casamino acids as substrates. The universal electron acceptor is elemental sulfur, however, polysulfides and thiosulfate are also utilized [1].

In this paper, we describe the complete genome sequence of *T. tenax* (strain Kra1, DSM 2078^T^), which gives new insights into the physiological versatility and regulatory potential of this organism.

So far, only 26 crenarchaeotal genomes, of which eleven belong to the genus *Sulfolobus* and four to the genus *Pyrobaculum* (according to NCBI, http://www.ncbi.nlm.nih.gov/genomes/lproks.cgi) have been sequenced, versus a total of 53 euryarchaeal genomes, two thaumarchaeal genomes (*Cenarchaeum symbiosum*, *Nitrosopumilus maritimus*) [3], [4] and two, not yet validly described and classified strains, i.e. *Nanoarchaeum equitans* [5], and Candidatus *Korarchaeum cryptofilum* [6] (www.genomesonline.org) [7]. In addition, the *T. tenax* genome is of special interest, since it is meanwhile adopted that the related *Thermoproteus neutrophilus* (strain V24Sta) obviously belongs to the genus *Pyrobaculum*. Therefore, *T. tenax* represents the first member of the genus *Thermoproteus* with available whole genome sequence information. Thus, the present study will not only contribute to unravel unique traits of this organism, but will also contribute to balance the disproportion between the known genomic content of *Crenarchaeaota* and *Euryarchaeota*. The here reported detailed genomic analysis, reveals new insights into the physiology as well as genetics and information processing of *T. tenax*. In addition to the previously suggested reductive TCA cyle [8], [9], all genes encoding enzymes of the novel dicarboxylate/4-hydroxybutyrate cycle [10] were identified, thus, raising questions about the activity of both pathways. In accordance with its autotrophic lifestyle, all pathways for the synthesis of the 20 proteinogenic amino acids were identified in *T. tenax*. Interestingly, the organism harbors the typical bacterial pathways for the complex branched chain and aromatic amino acid biosynthesis and in addition, archaeal routes, e.g. for proline biosynthesis.

Under autotrophic growth conditions *T. tenax* seems to gain energy by hydrogen oxidation via a single Iron-Nickel hydrogenase and sulfur reductase, which form a short electron transport chain probably mediated by quinones. Energy conservation under heterotrophic growth conditions seems to proceed via a membrane-bound electron transport chain and sulfur has been suggested as final acceptor. Interestingly, two complete operons encoding proteins of complex I (NADH:quinone oxidoreductase) were identified and the genome data give some evidence that either NADH or reduced ferredoxin can serve as electron donator. The presence of the three subunits for NADH binding and oxidation (Nqo1–3 or NuoEFG, NuoG gives ambiguous results) is so far rare for an anaerobic *Archaeum*. Beside the structurally unusual archaeal A_0_A_1_-ATP synthase, a membrane-bound pyrophosphatase seems to be involved in chemiosmosis. The biggest surprise, in respect to physiology, was the identification of all genes required for dissimilatory sulfate reduction and, indeed, growth in the presence of sulfate as terminal electron acceptor could be observed (unpublished data).

Protein transport in *T. tenax* seems to proceed via the “Sec translocase” secretion pathway as well as the twin arginine translocation (Tat) system. For ion and metabolite transport, as in most *Archaea* a PEP-dependent phosphotransferase (PTS) system is absent and *T. tenax* harbors about twice as much secondary transporter compared to ABC transporters. Information processing (i.e. replication, transcription, translation) in *T. tenax* resembles, like in all *Archaea*, the respective eukaryal counterparts. Interesting is the finding of four different TFB homologs in *T. tenax*. Multiplicity of general transcription factors is commonly found in *Archaea* and a function similar to sigma factors has been proposed previously [11]. In the *T. tenax* genome no extrachromosomal elements were identified. However, seven clusters of CRISPRs as well as Cas proteins were identified in the genome; the spacer sequences do not show similarity to archaeal viruses and plasmids, which are known to infect or transform *T. tenax*.

*T. tenax* harbors the archaeal gene core (157 genes) as well as all 234 *Crenarchaeota-*specific arCOGs as revealed by comparative genomic analyses. In the *Thermoproteales* lineage, 19 core gene families have been acquired specifically among those a ParA family ATPase and an actin-like protein. This is of special interest, since the ESCRT system, identified as the major system for cell division in *Archaea* [12], is missing in *Thermoproteales*. In addition, six *T. tenax* specific arCOGS were identified, which are absent in all other crenarchaeal genomes, and among those are genes involved in genetic competence and uptake of DNA (DprA, ComF), which have not been detected in *Archaea* before.

## Results and Discussion

### General genome features

The genome of *T. tenax* consists of a circular chromosome of 1,841,542 bp with an average G+C content of 55.1%. No extra-chromosomal elements remained after the genome sequence assembly. Analysis of the cumulative GC skew of the draft genome sequence was used in search for the origin of replication (http://mips.gsf.de/services/analysis/genskew); the genome sequence was subsequently reorganized, so that the global minimum of the GC skew marks the beginning of the genome sequence (bp 1). However, the only copy of a *cdc6* gene, which together with the global minimum of the GC-skew and the ORB-motif is supposed to be a marker for archaeal replication origins [13], is located far away at about 1.6 Mbp (TTX_1848), and the only conserved ORB-motif is located at position 58,820-58,094. Therefore, given the scattered distribution of these three elements, the location of the origin of replication stays uncertain.

Overall 2,051 predicted protein encoding open reading frames (ORFs) remained in the consensus gene set after manual deletion of small, most probably artificial ORFs, covering a total of 90.6% of the genome, which is, as in the closely related *Thermofilum pendens* (91%) only slightly higher than the values for most other sequenced *Crenarchaeota*, e.g. *Aeropyrum pernix* (89.1%), *P. aerophilum* (88%) or *Sulfolobus solfataricus* and *S. tokodaii* (85%). Only one copy for each rRNA gene, 5S (unlinked), 16S, and 23S rRNA, respectively, had been identified in the genome. As common for the *Crenarchaeota*, many of the 47 annotated transfer RNA genes contain an intron (see below and Table 1). Genes encoding the stable RNA components of RNaseP or the signal recognition particle (7S RNA) are absent, like in most other Thermoproteaceae (according to Rfam database (http://rfam.sanger.ac.uk/) [14]. About 75% of the predicted 2,051 protein coding sequences (1,552 ORFs), could be linked with a putative function, whereas most recent *Crenarchaeota* annotations name about 60% genes with predicted functions. Twenty-four percent (a total of 497) ORFs were assigned as (conserved) hypothetical or uncharacterized conserved proteins. Totally, 76.6% (1,572) of all predicted proteins were linked to COGs [15] and 95% (1,953) to arCOGs [16], which is slightly above average for crenarchaeotal genomes. Only about 4% (a total of 91) of the 2,051 predicted proteins appear to be unique for *T. tenax*.

**Table 1:** General genome features of *T. tenax*.

Genome size	1,841,542 bp	
G+C content	1,015,210 bp	55.13%
Coding region	1,669,147 bp	90.6%
***CRISPRs*** (total of 149 repeat modules)	7	
***Stable RNAs***		
23S rRNA	1 (1,700,676-1,703,699)	
16S rRNA	1 (1,699,104-1,700,606)	
5S rRNA	1 (1,298,635-1,298,753)	
tRNAs (13 with canonical introns)	46 (plus one pseudogene)	
***Predicted protein coding ORFs***	2,051 (1.1 per kb)	
Average ORF length	813 bp	
Average intergenic region	127.6 bp	
Predicted functions assigned	1,552	75.7%
(Conserved) hypothetical and uncharacterized conserved proteins	497	24.2%
No annotation	2	<0.1%
Assignment to COGs	1,572	76.6%
Assignment to arCOGs	1,953	95.2%
ORFs unique for *T. tenax*	91	4.4%
ORFs for proteins with signal peptides	56	2.7%
ORFs for proteins with transmembrane helices	412	20.1%

About 2.7% (a total of 56) of the predicted proteins possess a signal peptide. The fraction of transmembrane proteins (20.1%, a total of 412) is normal within the *Crenarchaeota*. No genes required for the usage of selenocysteine as 21^st^ amino acid were identified. Inteins could not be detected in any of the predicted proteins.

Genes involved in lipopolysaccharide (LPS) synthesis are frequently clustered in regions of microbial genomes that differ significantly from their average G+C content [17]. The function in *Archaea* is still unclear, since *Archaea* generally harbor no outer membrane (except *Ignicoccus hospitalis*; [18]) and LPS, commonly found in Gram-negative *Bacteria*. The *T. tenax* genome contains three extended regions of low G+C content (<47%, Table S1). Sixteen of the 23 genes encoded in the largest of these regions (region 3) have functions required for or linked to LPS synthesis, including nine type I/II glycosyltransferases, two polysaccharide biosynthesis proteins, two N-acetyl-glucosaminyl-phosphatidylinositol synthesis proteins, LPS-biosynthesis glycosyltransferase and a membrane protein involved in export of O-antigen. Low G+C region 1 encodes only the three subunits of an ABC transporter that might play a role in the transport of sugar monomers across the periplasm. For the LPS genes encoded in region 3, there is no evidence for a common origin *via* lateral gene transfer from a donor with low G+C content. Some of the genes in this cluster are most similar to homologs found in a variety of other *Archaea*, whereas others are most similar to bacterial homologs. Gene duplication in *T. tenax* as the source of the ten glycosyltransferases in this region can be excluded, because the encoded proteins share a higher degree of sequence similarity with homologs from other organism than between each other.

The largest protein in the genome, encoded by TTX_1887 (2,663 amino acids, corresponding molecular mass of 287 kDa), is a candidate for the S-layer protein, as it shows several of the required features: (i) the protein is rich in serine, threonine, and asparagine as putative glycosylation sites, (ii) it has an N-terminal signal sequence, and (iii) a C-terminal TM helix. Therefore, it is predicted to be anchored in the cytoplasmic membrane facing the environment [19], [20]. When using the NetNGlyc and NetOGlyc servers (http://www.cbs.dtu.dk/services/NetNGlyc/ and http://www.cbs.dtu.dk/services/NetOGlyc/) [21] for glycosylation prediction, five putative O-glycosylation site are predicted and multiple N-glycosylation sites. The genome contains seven (low copy number, 2–5 copies) repeats longer than 300 bp with more than 95% sequence conservation. The longest of these repeats is a pair of cobyrinic acid a,c-diamide synthase genes, *cbiA* (TTX_0412 and TTX_1195); another pair contains putative cobalamin adenosyltransferases (TTX_0290 and TTX_1504). Five ORFs (TTX_0813, TTX_0867, TTX_1864, TTX_1903, TTX_1904) encoding putative transposases or fragments of inactivated transposases are identified in the *T. tenax* genome, indicating the rare presence of genetically mobile IS-elements.

In the genome of *T. Tenax*, seven clusters of regularly interspaced short palindromic repeats (CRISPRs) could be identified (coverage 0.5%; Table S2). In general, *Archaea* show in comparison to *Bacteria* very extensive CRISPR clusters and have a highly divergent gene organization of the strictly associated *cas* genes [22], [23], [24]. The CRISPR/Cas system is supposed to guide antiviral defence by sequence similarity between spacer and phage genome, but also to limit horizontal gene transfer by preventing conjugation and plasmid transformation [25], [26], [27].

The five type I repeat clusters are significantly longer than the two type II clusters (Table S2) and show a larger variation in the lengths of the spacer sequences: 37–55 bp *versus* 41–48 bp. The two types of clusters also differ significantly in the length of their leader sequences. Leader sequences of type I are shorter than those of type II (317–327 bp *versus* 613–624 bp) [23], and also show a higher degree of sequence similarity between each other. The spacer sequences between the repeat units in CRISPRs are considered to derive from extra-chromosomal elements [24], [28], but homology searches revealed no significant matches between the spacer sequences of *T. tenax* CRISPRs and archaeal viruses and plasmids, which are known to transform *T. tenax* (TTV1, TTSV1, and PSV) [29], [30], [31]. The conserved genes *cas1* to *5* and the gene of a putative HD-domain superfamily hydrolase (TTX_1254) are clustered between CRISPR 5 and CRISPR 6 and occur near one of the organisms̀ repeat clusters. Thus, the CRISPR organisation of *T. tenax* corresponds to the *A. pernix* subtype [22]. Like in other *Crenarchaeota*, these genes are associated with three genes belonging to COG4343 (TTX_1248), COG1857 (TTX_1251), and COG0640 (TTX_1249).

All general genome features are summarized in Table 1. Table S8 provides all identified *T. tenax* genes including gene IDs, functional assignments as well as the GenBank GI accession numbers for BLASTP best hits against NCBI Non-redundant database (e-value cutoff 0.001).

### Central metabolism

#### Central carbohydrate metabolism (CCM)

**Figure 1:**
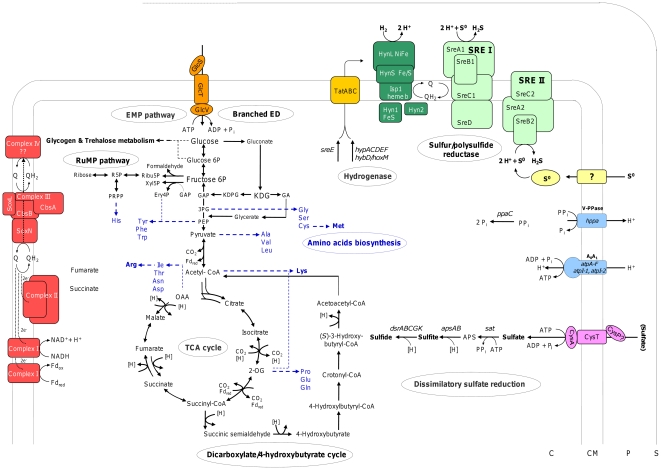
Central metabolic pathways of *T. tenax*. Pathways for carbon metabolism (glucose uptake, glucose metabolism, carbon dioxide fixation, amino acid biosynthesis) and energy metabolism (electron transfer chains, A_1_A_0_-ATP synthase, PP_i_ase, sulfate reduction) are depicted. The amino acid pathways are indicated in blue, dashed lines in order to better distinguish from the other carbon and energy metabolic pathways. Assumed electron transport is given in dotted lines and trehalose as well as glycogen metabolism are implied in black, dashed lines. For abbreviations and gene IDs see respective text sections.

The CCM of *T. tenax* has been studied in great detail, and genome analysis in combination with biochemical studies revealed the presence of a modified reversible EMP pathway as well as an unusual branched ED pathway for sugar degradation (Figure 1) (for review see [32]). Organic substrates are completely oxidized to CO_2_ via the oxidative TCA cycle [33], whereas CO_2_-fixation under autotrophic growth conditions has been assumed to proceed via the reductive TCA cycle (Figure 1) [8], [9], [32]. However, recent studies proposed a novel dicarboxylate/4-hydroxybutyrate cycle for autotrophic CO_2_ fixation as common CO_2_ fixation mechanism within autotrophic members of the *Thermoproteales* [10], [34], [35]. Surprisingly, in the genome of *T. tenax* all required genes for a functional dicarboxylate/4-hydroxybutyrate cycle could be identified (Figure 1; Table 2). Therefore, experimental analyses have to be awaited in order to elucidate the role of both pathways in CO_2_ fixation in *T. tenax*. Recent studies revealed that the conventional oxidative pentose phosphate pathway (OPPP), which is essential for the generation of pentoses, reducing power (NADPH) and erythrose 4-phosphate (E4P) for amino acid biosynthesis, is generally absent in *Archaea* [36], [37]. Beside the non-oxidative pentose phosphate pathway (NOPPP), the so-called reversed ribulose monophosphate (RuMP) pathway has been shown to provide pentoses for anabolic purposes in most *Archaea* [36], [37]. The pathway is characterized by the two enzymes 3-hexulose-6-phosphate isomerase (PHI) and 3-hexulose-6-phosphate synthase (HPS) that catalyze the isomerization of fructose 6-phosphate (F6P) to 3-hexulose-6-phosphate and the reversible cleavage into formaldehyde and ribulose 5-phosphate (Ru5P; Figure 1). The HPS-PHI fusion proteins from *Pyrococcus horikoshii* [38] and *Thermococcus kodakaraensis* [39] have recently been characterized. In the genome of *T. tenax* two single ORFs, TTX_1521 and TTX_1049 have been identified, which code for a single HPS and PHI, respectively [40].

**Table 2:** *T. tenax* candidate genes coding for the key enzymes involved in the dicarboxylate/4-hydroxbutyrate cycle.

Enzyme	*T. tenax* ORF ID	Gene ID best hit aa identity and e-value
PEP-carboxylase (*pepC*)	TTX_1107	Tneu_0418 (100%, 0.0)
		Igni_0341 (91%, 6e-60)
Succinyl-CoA reductase	TTX_1101	Tneu_0421 (99%, 0.0)
Succinic semialdehyde reductase	TTX_1106	Tneu_0419 (100%, 5e-138)
4-Hydroxybutyrate-CoA ligase (AMP-forming)	TTX_1100	Tneu_0420 (82%, 0.0)
4-Hydroxybutyryl-CoA dehydratase	TTX_1102	Tneu_0422 (100%, 0.0)
Crotonyl-CoA hydratase/(*S*)-3-Hydroxybutyryl-CoA DH	TTX_1028	Tneu_0541 (99%, 0.0)
		Igni_1058 (99%, 1e-160)
Acetoacetyl-CoA β-ketothiolase	TTX_0886	Tneu_0249 (99%, 1e-166)
		Igni_1401 (99%, 2e-104)

The corresponding e-values derived from blastp analyses of the *T. neutrophilus* (Tneu_) and *I. hospitalis* (Igni_) candidates involved in the cycle in these organisms [10], [34], [35] are given.

For the biosynthesis of the aromatic amino acids erythrose 4-phosphate (E4P) is required as precursor, which is formed from F6P and glyceraldehyde 3-phosphate via transketolase (Figure 1). In *T. tenax* two ORFs encoding the N- and the C-terminus of transketolase (*tktA*, *tktB*; TTX_1754, TTX_1753) have been identified, which cluster with genes involved in the synthesis of the aromatic amino acids.

#### Amino acid biosynthesis

From the genome data it can be assumed that *T. tenax* possesses pathways for the biosynthesis of all 20 proteinogenic amino acids (Figure 1; Table S3). Most of the genes involved in amino acid biosynthesis are organized in large gene clusters, e.g. genes involved in histidine, aromatic and branched chain amino acid synthesis (Table S3). Interestingly, most of the reconstructed pathways resemble the common pathways of the *Bacteria* (e.g. *Escherichia coli*, *Bacillus subtilis*) and the *Eucarya* (e.g. yeast). For example, all genes encoding enzymes required for the complex biosynthesis of tryptophane, tyrosine and phenylalanine from phosphoenolpyruvate and E4P *via* shikimate and chorismate, could be identified in the *T. tenax* genome (Table S3). There is no evidence for the existence of the recently described archaeal aspartate-semialdehyde pathway [41].

Also, all genes encoding the enzymes for the conventional synthesis of the branched chain amino acids valine, leucine and isoleucine, could be found in *T. tenax* (Table S3). Interestingly, this is contrast to the closely related *T. neutrophilus* [42] as well as other *Archaea* [43], [44], which use the citramalate cycle. In many *Archaea*, most of the genes encoding enzymes of the conventional pathway for proline synthesis from glutamate (e.g. glutamate 5-kinase; EC 2.7.2.11) are absent, and it has previously been shown that varying pathways for the synthesis of proline are used [45], [46], [47]. From the genome data two possible routes for the synthesis of proline could be proposed in *T. tenax*: (i) Biosynthesis from glutamate via 1-pyrroline-5-carboxylate dehydrogenase (*putA*, EC1.5.1.12; TTX_1787), which catalyzes the formation of γ-glutamic semialdehyde leading to pyrroline-5-carboxylate, and pyrroline-5-carboxylate reductase (*proC*, EC1.5.1.2; TTX_1730) converting pyrroline-5-carboxylate into proline. (ii) Cyclization of the non-proteinogenic amino acid L-ornithine catalyzed by the ornithine cyclodeaminase (OCD, *arcB*, EC 4.3.1.12; TTX_2070, TTX_0618), like it is described for *M. jannaschii* [45].

The genome data further indicate that arginine is most likely not synthesized from glutamate *via* the conventional route due to the lack of a gene encoding acetylglutamate synthase (EC 2.3.1.1) catalyzing the first step of arginine synthesis. However, alternative pathways for arginine synthesis either from carbamoyl-phosphate (via ornithine carbamoyltransferase (*argF*, EC 2.1.3.3; TTX_0091), argininosuccinate synthase (*argG*, EC 6.3.4.5; TTX_0123) and argininosuccinate lyase (*argH*, EC 4.3.2.1; TTX_0467) in the urea cycle, or from aspartate (via argininosuccinate synthase (*argG*, EC 6.3.4.5; TTX_0123) and argininosuccinate lyase (*argH*, EC 4.3.2.1; TTX_0467) could be identified in *T. tenax*.

For methionine synthesis, *T. tenax* most likely uses a pathway starting from homocysteine *via* methionine synthase (*metE*, EC 2.1.1.14; TTX_1021), like it is also described for *M. thermautotrophicus* [48] and supposed for *M. jannaschii* [49].

Like in many other *Archaea*, e.g. *T. neutrophilus* [42], *P. aerophilum* [50], *I. hospitalis* [44], and lower *Eucarya* [51], lysine synthesis in *T. tenax* proceeds via the aminoadipate pathway from 2-oxoglutarate and acetyl-CoA. The complete set of genes has been identified in the *T. tenax* genome (Table S3), whereas four of nine genes encoding enzymes required for the alternative synthesis of lysine via the widespread diaminopimelate pathway [52] are missing.

All but one gene (*hisB*) encoding enzymes for histidine synthesis from phosphoribosyl pyrophosphate (PRPP) have been identified in the *T. tenax* genome (Table S3). However, the lack of histidinol phosphatase (*hisB*, EC 3.1.3.15) has also previously been reported in other *Archaea* (e.g. *Archaeoglobus fulgidus*, *M. thermautotrophicus* [53], [47], [54] and a substitution of histidinol phosphatase by HD superfamily phosphohydrolases [55] has been suggested [47]. The *T. tenax* ORF TTX_1708 is coding for a phosphohydrolase of the HD superfamily (COG1078).

### Energy metabolism

#### Chemolithoautotrophic growth

Like other obligatory sulfur reducers (e.g. *Acidianus ambivalens*, *Pyrodictium occultum*, *Pyrodictium abyssi* and *T. neutrophilus*), *T. tenax* gains energy from anaerobic H_2_ oxidation with sulfur as terminal electron acceptor (hydrogen-sulfur autotrophy).

**Figure 2:**
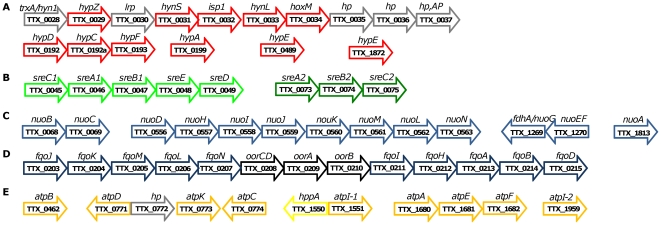
Gene organization. Genes encoding Iron-Nickel hydrogenase (including accessory genes) (**A**), sulfur/polysulfide reductases (**B**), and gene clusters of the two sets of complex I (**C** and **D**) as well as A_1_A_0_-ATP synthase (**E**) from *T. tenax* are shown. The annotated ORFs and their orientation is indicated by arrows (not to scale), the gene names and the respective gene IDs are given. **A**: *trxA/hyn1* – Thioredoxin/Rieske ferrdoxin; *hypZ* – [Ni,Fe]-hydrogenase maturation factor; *lrp* – Transcriptional regulator, Lrp/AsnC family; *hynS* – [Ni,Fe]-hydrogenase I, small (41 kDa) subunit; *isp1*- [Ni,Fe]-hydrogenase, cytochrome b subunit (29 kDa); *hynL* - [Ni,Fe]-hydrogenase I, large (66 kDa) subunit; *hoxM* - [Ni,Fe]- hydrogenase maturation factor for HynL; *hp* – hypothetical protein; *hp,AP* - phosphodiesterase/nucleotide pyrophosphatase, AP (anchored protein) superfamily; *hypDCFAE*, hydrogenase expression/formation proteins. **B**: *sreA1/2*, sulfur reductase large 100 kDa subunit (Mo-FeS protein); *sreB1/2*, sulfur reductase FeS subunit; *sreC1/2*, membrane protein; *sreD,* FeS electron transfer protein; *sreE,* reductase assembly protein. **C** and **D**: *nuoABCDEFGHIJKLMN*, subunits of the NADH:quinone oxidoreductase (complex I); *fdhA/nuoG*, fdhA/NADH-oxidizing subunit; *fqoAB-* and *fqoHIJM-N* subunits of the second set of NADH:quinone oxidoreductase (complex I); *oorA-B-CD*, 2-oxoacid oxidoreductase subunits. **E**: *atpABCDEI-1I-2* subunits of the membrane-bound A_1_A_0_- ATP synthase; *hp* hypothetical protein; *hppA*, membrane-bound proton-translocating pyrophosphatase (vacuolar-type H^+^-pyrophosphatase).

Hydrogen oxidation and sulfur reduction require the presence of a hydrogenase and a sulfur reductase. The *T. tenax* genome contains a single set of genes encoding the Iron-Nickel hydrogenase subunits including the large NiFe subunit HynL (TTX_0033), the smaller FeS subunit HynS (TTX_0031), and the membrane anchor protein Isp1 (*hemeB*; TTX_0032). The corresponding accessory genes required for the maturation of HynL (*hypACDEF* TTX_0192, TTX_192a, TTX_0193, TTX_0199; TTX_0489; TTX_1872; Fig. 2A) and two maturation proteases *hybD/hoxM* (TTX_0029, TTX_0034) are scattered over the genome. The presence of a single set of hydrogenase genes suggests that the gene products are responsible for hydrogen uptake during chemolithoautotrophic growth (Figure 1).

Similar to the *Archaea A. ambivalens, P. abyssi* and the bacterium *Wolinella succinogenes* [56], [57], [58], the hydrogenases form short and rather simple electron transport chains with sulfur or polysulfide reductases (SR/PSR) in *T. tenax* (Figure 1). One pentacistronic operon in *T. tenax* shows exactly the same gene composition as the *A. ambivalens* SR operon with a 30-50% amino acid identity of the reading frames. Both operons comprise genes encoding the MoPterin (*sreA1*) and the FeS subunits (*sreB1*), a membrane anchor protein (*sreC1*), a polyferredoxin of unknown function (*sreD*), and a system-specific chaperone (*sreE*) similar to nitrate reductase maturation proteins NirD (Figure 2B; TTX_0045-0049). The presence of a TAT motif in the large MoPterin and the FeS subunit suggests the export of these subunits across the membrane. In addition to the pentacistronic SR operon, a second, tricistronic operon with SR homologs (*sreA2-sreB2*-*sreC2*; TTX_0073-0075; Figure 2B) could be identified. In contrast to the previously described operon, TAT motifs in *sreA2* and *sreB2* are absent, suggesting a cellular orientation of these subunits (Figure 1).

Electron transfer between hydrogenase and SR is most probably mediated by quinones (Figure 1), since no indication for the presence of c-type cytochromes was found. The TAT motif in the hydrogenase FeS and the SR MoPterin proteins suggests that the catalytic subunits are oriented outwardly, extending into the “quasi-periplasmic space” (Figure 1) [59], [60]. Therefore, the question arises, how a proton motive force is generated during hydrogen oxidation and sulfur reduction. We assume that a Q cycle is in operation facilitating the uptake of protons in the cytoplasm during quinone reduction by the hydrogenase and the release into the quasi-periplasm upon re-oxidation by the SR.

*Chemoorganoheterotrophic growth. T. tenax* completely oxidizes organic compounds to CO_2_ via the oxidative TCA cycle [8], [33]. Energy is conserved by a membrane-bound electron transport chain and sulfur has been suggested as final electron acceptor (sulfur respiration). The NADH:quinone oxidoreductase (complex I) is encoded by minimum of 14 genes in aerobic microorganisms (*nuoA-N* or *nqo1-14*). [61]. Complex I genes were found in many genomes of anaerobic *Archaea* including *Archaeoglobus* and also in several methanogens. Only 11 of the 14 subunits are conserved in most of the anaerobes, while the others are replaced by non-homologous ferredoxin or F_420_-oxidizing subunits. The three other subunits (Nqo1-3 or NuoEFG) catalyze NADH binding and oxidation in complex I of aerobes.

Surprisingly, *T. tenax* has two presumably complete sets of complex I genes (Figure 1), one of which seems to include the NADH-binding subunits (*nuoA-N*). The *nuo* genes are spread over four operons across the genome (TTX_1813; TTX_0068-0069; TTX_0556-0563; TTX_1269-1270; Figure 2). 13 out of 14 of these genes can be assigned unambiguously, only one of the NADH-oxidizing subunits (NuoG) gives ambiguous results. The second set of complex I genes is located at a single site in the genome (*fqo/oor*; TTX_0203-0215), however, it includes uncommon subunits. The *fqo* genes are strikingly similar to the F_420_H_2_:quinone oxidoreductase known from several methanogens and from *Archaeoglobus fulgidus*. 10 out of 12 *A. fulgidus fqo* genes are conserved in *T. tenax* (Figure 2), while the F_420_-oxidizing *fqoF* subunit is missing in accordance with the fact that the organism does not use this cofactor. In the middle of this region three 2-oxoacid oxidoreductase genes are found (*oorA-D*, TTX_0208-0210; Figure 2). This unprecedented observation raises the question, whether these *oor* genes encode a separate soluble enzyme with OOR activity or, whether the protein replaces the substrate-oxidizing subunits in the membrane-bound complex I to funnel electrons directly from the oxidative 2-oxoacid decarboxylation into the quinone pool.

Succinate dehydrogenase (complex II; Figure 1) provides another electron entry point in the respiratory chain. One complete set of *sdh* genes, including membrane anchor proteins, is present (TTX_0861-0864) as well as additional genes encoding a second flavoprotein and FeS subunit, respectively (TTX_1104-1105). It cannot be convincingly decided, without biochemical analyses, which of the genes encodes SDH, present in the TCA cycle, and whether, some of these genes might also encode the fumarate reductase [8].

In addition, genes encoding an analog of the *bc1* complex (complex III; Figure 1) are also present. The genes are arranged in the same order as in *C. maquilingensis*: One bicistronic operon encodes a Rieske protein (SoxL, TTX_0319, the only Rieske protein or Rieske ferredoxin in the genome) and a b-type cytochrome, respectively (SoxN, TTX_0318), while the other operon is transcribed in the opposite direction from the same promoter region and encodes another *b*-type cytochrome (CbsA, TTX_0320) and a membrane protein of unknown function (CbsB, TTX_0321). This *bc1*-analogous complex was previously identified in *S. acidocaldarius* and in *A. ambivalens* [62], [63], and supposedly transfers electrons from quinol to an unknown high-potential electron carrier in the Sulfolobales, which finally transfers them to the terminal oxidase. A *bona fide* terminal oxidase was not identified in the *T. tenax* genome, however, two paralogous copies of the subunit I of a *bd* oxidase are present (*cydA*, TTX_0142 and TTX_0143). Many of the essential residues are conserved in TTX_0142 [64], [65], and therefore, the questions arise, whether this is indeed an oxygen-reducing enzyme and whether it is part of an oxygenic electron transport chain. Many anaerobic *Archaea* carry either this combination of terminal oxidase genes or alternatively, homologs of subunits I and II of these enzymes [64], [65]. Their *in vivo* structure and function remains to be elucidated, however, it is tempting to speculate that they might play a role in *T. tenax* under microaerobic growth conditions, although *T. tenax* is described as obligate anaerobe. The presence of multiple ferredoxin genes is characteristic for many *Archaea*. At least six different *fdx* genes are identified in *T. tenax* (TTX_0439, TTX_0681, TTX_0731, TTX_0985, TTX_1318, TTX_2019) either encoding 4Fe4S or 7-8Fe ferredoxins. They have been implicated in oxygen protection, in electron transfer between organic substrates and electron transport chains and as general redox carrier in the absence of c-type cytochromes. The involvement in electron transfer is supported by the presence of multiple genes encoding for example 2-oxoacid:ferredoxin oxidoreductases or aldehyde:ferredoxin oxidoreductases [8]. The link to membrane-bound electron transport chains could be provided by two sets of electron transfer flavoprotein complex (*etf*) genes encoding oxidoreductases that shuffle electrons to or from unknown membrane-bound proteins and the ferredoxin:quinone oxidoreducase subunits of complex I (see above) [66].

Surprisingly, a complete set of genes required for dissimilatory sulfate reduction could be identified in the *T. tenax* genome. This set comprises *sat* encoding an ATP sulfurylase (TTX_0441), *apsAB* encoding the APS reductase (TTX_0428-0429), and *dsrABCGK* (TTX_1185-1188, TTX_1191) encoding the dissimilatory siroheme sulfite reductase including a so far unidentified membrane anchor protein (Figure 1). The functionality of the dissimilatory sulfate reduction has been confirmed by chemoorganotrophic growth of *T. tenax* on sulfate as electron acceptor (Hensel, unpublished data).

#### Chemiosmosis

An archaeal, membrane-bound A_0_A_1_-ATP synthase is present in *T. tenax* (for review see [67]). As reported for *Crenarchaeota*, the subunits are spread in the genome and beside single genes two gene clusters were identified (Figure 2). Interestingly, *T. tenax* harbors two copies of *atpI* (*atpI-1*, *atpI-2*) coding the subunit “a” of A_0_, which forms the stator in archaeal ATPases (Figure 1). This is so far unique within the *Archaea*, but the meaning of this gene duplication is yet unknown. Sequence signatures of the membrane integral A_0_-subunit c (*atpK*) suggest that protons, rather than Na^+^, are translocated over the membrane by the *T. tenax* ATPase [68]. The presence of a membrane-bound pyrophosphatase (*hppA*, TTX_1550) indicates that the hydrolysis of PP_i_ contributes, at least partially, to the membrane potential, as shown for the vacuolar-type membrane pyrophosphatase of *P. aerophilum* (V-PPase, PAE1771) [69], [70]. Interestingly, TTX_1550 encoding the respective *T. tenax* homolog (78% aa identity, (560/717)), is found in a divergent organization with *atpI-1* (TTX_1551; Figure 2), suggesting a regulatory function. Additionally, a soluble, cytoplasmic pyrophosphatase is present in *T. tenax* (*ppA*, TTX_0388), which is supposed to have an important function to drive biosynthetic processes such as DNA synthesis.

### Protein and ion transport

#### Protein transport

Next to the essential general secretion system, Sec61αβγ (TTX_1416, TTX_1720, TTX_1808), *T. tenax* possesses the twin arginine translocation (Tat) system (*tatA*, TTX_2052, *tatC*, TTX_1059 and *tatD*, TTX_0685), which transports proteins in their fully folded state across membranes. As in other *Archaea*, *tatB* is not present in the *T. tenax* genome [71]. Possible Tat substrates were predicted using the TatFind program (Table S4.a) [72]. They include HynS, SreA1, ornithine carbamoyltransferase, a hypothetical protein, as well as SoxL, ABC-type branched-chain amino acid transport system (periplasmic component) and formate dehydrogenase (alpha subunit). Three operons (TTX_0962-0973, TTX_1130-1136, TTX_0887-0898) are identified, which might constitute type IV pili (TP4) assembly systems. Bacterial type IV pili are involved in a variety of functions such as twitching motiliy, cell-cell contacts, adherence and DNA uptake [73]. All three operons contain ATPases, which are known to be essential for the assembly processes of TP4. The TTX_0962-0973 and the TTX_1130-1136 operon contain next to the ATPase pilin like proteins, which might either function in the transport process or might be subunits of a pilus. However, *T. tenax* does not seem to contain a flagellum operon as typical flagellar accessory proteins as FlaI, FlaH or FlaJ are missing.

To be targeted to one of these systems, precursor proteins are equipped with signal peptides. In *T. tenax* 70 proteins contain a signal peptide (∼3.4%). The majority (48 signal peptides) are class 1 signal peptides that target the protein to the Sec translocase and they have been identified using SignalP [74]. Whereas seven proteins contain putative Tat dependent signal peptides (mentioned above, Table S4.a), 15 exhibit a type IV pilin like signal peptide predicted by the program FlaFind (Table S4.b) [75] and might be pilin subunits. *T. tenax* contains a clear leader peptidase homolog (TTX_1710), involved in the processing of sec dependent signal peptides. A possible candidate for a type IV prepilin peptidase was also identified (TTX_0979).

#### Ion and metabolite transport

A total of 412 proteins, 20.1% of the predicted proteome of *T. tenax* is localized in the membrane. Of these 412 proteins, 133 proteins (6.5% of the total amount of protein coding ORFs) can be classified as transporters (Table S5.a; for classification see I. Paulsen's transport database, http://www.membranetransport.org/) [76]. No indications for the presence of PEP-dependent phosphotransferase (PTS) systems were observed in the *T. tenax* genome, which is in accordance with most *Archaea* investigated so far (with the only exception of *Haloarcula marismortui*, *T. pendens* and *Haloquadratum walsbyi*, the latter only harbors enzyme I and HPr) [77]. The distribution of the different transport classes is comparable to the one from *S. solfataricus*. Both do have two times more secondary transporters than ATP-dependent transporters. About half of all *T. tenax* transport proteins (66 of 133) share highest similarity with transporters from *P. aerophilum*, whereas 32 are closest related to proteins from *S. solfataricus*. Analysis of the 15 substrate binding proteins of ABC transporters of *T. tenax* (Table S5.b) showed that only two have a N-terminal “bacterial” like sec-dependent signal peptide and are subsequently anchored by a C-terminal transmembrane domain to the membrane (N-terminus outside, C-terminus inside). The transmembrane domain is preceded by a ST-linker, a stretch of serine or threonine residues [78]. These linker regions are often known to be O-glycosylated at the serine or threonine residues. *T. tenax* binding proteins are glycosylated as they can be isolated by ConA (lectin) affinity chromatography (four binding proteins were identified by mass spectroscopy; Table S5.b), which is specific for terminal mannose residues. The majority of the *T. tenax* binding proteins has an N-terminal transmembrane domain followed by the ST (or SQ) linker (resulting in N-terminus inside, C-terminus outside). However, the type IV pilin-like signal peptide, identified in *S. solfataricus*, as well as the cysteine containing consensus motif implying lipidation in *Euryarchaeota* is absent [79]. Therefore, in *T. tenax*, similar to *P. aerophilum*, it is not clear, whether the binding proteins are N-terminally processed. Most probable the N-terminal transmembrane domain is used to anchor the binding protein to the membrane, which is supported by the position of the ST-linker.

### Genetics and Information Processing

#### Replication

The DNA replication machinery of *T. tenax* conforms to the archaeal norm by resembling that of Eucarya [80]. *T. tenax* encodes a single candidate initiator protein (TTX_1848) that is homologous to eucaryal Orc1 and Cdc6. Archaeal Cdc6 has been shown to contact the MCM helicase (TTX_0274). MCM acts to unwind DNA, whereupon the exposed single stranded DNA is coated by a single strand binding protein. Interestingly, neither *T. tenax* nor the closely related *Pyrobaculum ssp.* possesses obvious homologs of canonical SSBs. However, a recent study has identified a novel single-stranded binding protein, CC1, in *T. tenax* (TTX_1853/1420 (two genes with identical gene products) and TTX_0308) [81]. Whether CC1 performs the roles of canonical SSBs in the replication process remains to be determined. Archaeal primase is a heterodimer and both subunits are conserved in *T. tenax* (TTX_0579 and TTX_1586). Recent work has suggested that archaeal primase may be coupled to the progression of the MCM helicase *via* the bridging action of the GINS complex [82]. In this light, it may be significant that one of the two *T. tenax* GINS homologs (TTX_0578, GINS15) is encoded within an operon with the catalytic subunit of primase, which is found in many, but not all, *Archaea* [82]. Once the primer is synthesized, it is extended by the replicative DNA polymerase. *T. tenax* encodes three members of the family B DNA polymerases (TTX_0168, TTX_1461 and TTX_1917). In *Archaea* and *Eucarya* the attachment of Polymerase to their template is conferred by PCNA. Although *Eucarya* and *Euryarchaeota* generally have a single PCNA homolog that forms a homotrimer, the *Crenarchaeota* encode two or more PCNA subunits. *T. tenax*, like *P. aerophilum*, has two PCNA homologs (TTX_0580 and TTX_0869). Whether these form homo- or heteromultimers in *T. tenax*, is currently unknown. PCNA requires an additional factor, RFC, to load it on DNA. Archaeal RFC is normally a pentamer with one large subunit in complex with a homotetramer of a small subunit. *T. tenax* possesses homologs of both, the large and small subunit encoded within an operon (TTX_1850-1851) and, moreover, the ORF TTX_1485 is coding for a second homolog of the small subunit in *T. tenax*. The organism possesses a number of topoisomerases; reverse gyrase TTX_1984, a type 1A topoisomerase III homolog (TTX_1447) and a type 2 topoisomerase TopoVI. The latter typically contains two subunits, A (TTX_0746) and B. In *T. tenax*, the B subunit appears to be split into two halves (TTX_0744 and TTX_0745).

#### Transcription

The basic transcription apparatus of *Archaea* resembles the basal eucaryal RNA polymerase II system including homologs to the general transcription factors TATA-box binding protein (TBP), transcription initiation factor IIB (TFB in *Archaea*), and the alpha-subunit of transcription initiation factor IIE (TFE) [84], [85].

Clustering of genes encoding proteins of the basal transcription and translation machineries is a general feature of ‘prokaryotic’ genomes. Two separate gene clusters coding for RNA polymerase subunits and ribosomal proteins are conserved within the archaeal domain. The first one encompasses genes coding for the catalytic subunits (*rpoB*, *rpoA1*, and *rpoA2*) as well as subunit H, the second one encompasses the two assembly subunits (*rpoD* and *rpoN*) [86]. In *T. tenax*, *P. aerophilum*, and *T. pendens* the two gene clusters are fused and this organization might facilitate assembly of the RNA polymerase. Multiplicity of general transcription factors is commonly found in *Archaea*. Studies in *Halobacterium* NRC-1 revealed specific regulons for different TFB paralogs [87]. For TFB3 of *S. solfataricus* an activation of transcription by interaction with the ternary complex (DNA, TFB1, TBP) has been demonstrated [88]. The genome of *T. tenax* harbours single homologs for *tbp* (TTX_0178) and *tfe* (TTX_1936), but four *tfb* homologs (*tfb1*, TTX_1484, *tfb2*, TTX_2085, *tfb3*, TTX_1929, and *tfb4*, TTX_1732). TFB1 exhibits highest overall sequence similarity to the characterized TFB homologs of *Sulfolobus shibatae* (AAA81380) and *S. acidocaldarius* (AAF18139) [89], [90]. Like TFB3 of *S. solfataricus* the *T. tenax* TFB3 lacks one cyclin fold. Next to a classical homolog of transcription elongation factor S (TTX_0581), an additional paralog to transcription elongation factor S, lacking the conserved C-terminus required for stimulation of the intrinsic endonuclease activity of RNAP, was found in all genomes of the *Thermoproteaceae*: *T. tenax* (TTX_0711), *P. aerophilum* (PAE3480), and *C. maquilingensis* (Cmaq_0787).

#### Translation

*T. tenax* was found to contain one RNA operon comprising 16S and 23S RNA as well as a separate 5S RNA gene (Table S6.a). The *T. tenax* genome contains a full complement of 46 tRNA predictions, plus one apparent tRNA pseudogene [91], [92]. A total of 28 genes possess introns at non-canonical positions (10 tRNAs have two introns and one has three introns; http://gtrnadb.ucsc.edu/Ther_tena/Ther_tena-summary.html) Table S6.a).

With the only exception of L41e, which shows some uncertain distribution in *Archaea*, all conserved ribosomal proteins in the hitherto known archaeal genomes are present in *T. tenax* (Table S6.b). Interestingly, unique to *T. tenax* within the *Archaea*, an exact duplication of the ribosomal gene (S30e) is found (TTX_0151 and TTX_0161).

In contrast to *Bacteria* and most *Archaea*, which harbor large clusters of genes coding for ribosomal proteins (e.g. str locus containing the S10 - spc – alpha operons; [93]), the genome of *T. tenax* is characterized by rather short clusters with only up to five genes, a feature which seems to be typical for the members of the *Thermoproteaceae* (*T. tenax, P. aerophilum, P. islandicum, C. maquilingensis*). Aminoacyl tRNA synthetase genes for 20 amino acids were found in the genome (Table S6.c). No indications could be observed for the cotranslational incorporation of selenocysteine or pyrrolysine. Thus, all amino acids including asparagine and glutamine seem to be incorporated by direct acylation of the tRNA.

### Comparative genomics and phylogenetic position of *T. tenax* within the *Crenarchaeota*

**Figure 3:**
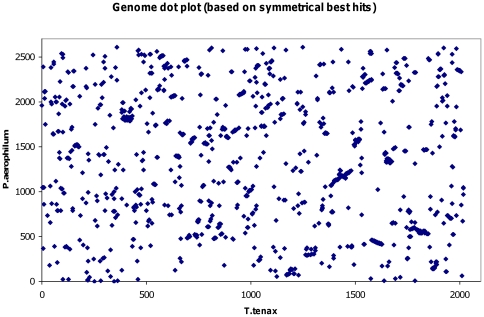
Genome dot plot comparison of *T. tenax* and *P. aerophilum*. CDSs in genomic order were tested for colinearity between the two genomes of *T. tenax* and *P. aerophilum*. Each point represents a matching pair of orthologs with an e-value of <1×e^−15^ (for approach see material and methods). The calculations yielded a value for C (colinearity factor) of 280. The comparison of bacterial genomes of similar size yielded values in the range of 18 (*Helicobacter pylori* J99 *vs H. pylori* 26695) to 238 (*Helicobacter acinonychis vs Wolinella succinogenes*) [119]. The very limited synteny between the genomes of *T. tenax* and *P. aerophilum* that keeps only the gene order of local islands intact, suggests major genomic rearrangements after their divergence from the common ancestor.

The phylogenetic position of *T. tenax* within the *Crenarchaeota* as a sister group of ‘*Pyrobacula’*, the group that contains all known *Pyrobaculum* species and *T. neutrophilus*, is confirmed by the 16S rRNA sequences (ARB-Living-Tree project) [94] and the phylogenetic tree based on three subunits of RNA polymerase (Figure S2). Although, thus, *T. tenax* is phylogenetically clearly separated from the genus *Pyrobaculum*, as a group, *T. tenax* and the *Pyrobacula*, can be clearly separated from the deeper branching *Thermoproteaceae* with *C. maquilingensis* and *Thermofiliaceae* with *T. pendens.* Despite the closer relationships between *T. tenax* and *P. aerophilum* within the *Thermoproteaceae*, the synteny in these genomes is minimal, suggesting extensive gene rearrangement, which has occurred after their divergence from the common ancestor (Figure 3).

We compared the protein complement of *T. tenax* to the database of clusters of orthologous groups developed specifically for archaeal genomes, arCOGs [16] (database update in preparation). We assigned 1,953 (95%) of the proteins to 1,604 arCOGs; the coverage is comparable to that observed for closely related *Pyrobaculum* species [16]. Overall, the gene content of *T. tenax* is typical of *Crenarchaeaota*. It preserved the intact 157 gene core shared by all archaeal genomes and additional 42 gene families that are missing only in the smallest *Archaeum*, i.e. *N. equitans*. *T. tenax* has not lost any of the 234 arCOGs present in all *Crenarchaeota*, including nine that are not present in any euryarchaeal genomes. The latter set includes five genes that are shared with *Eucarya*: the recently described small RPB8 subunit of DNA-directed RNA polymerase (TTX_1930) [95], a Zn-finger containing protein, an apparent transcription elongation factor 1 ortholog (TTX_1715), and ribosomal proteins S25e, S26e and S30e (TTX_0177, TTX_0164, TTX_0151 and TTX_0161 (Table S7). The conserved gene core of the *Thermoproteales* lineage consists of 607 arCOGs and representatives of only five, i.e. arCOGs 1304, 975, 5463, 921, 5461, are missing specifically in *T. tenax* (Table S7). Among the core gene families, 19 are not present in other crenarchaeal genomes, including five that are shared only with the deep-branching *Archaeum K. cryptofilum* and nine, which are so far unique for *Thermoproteales* and must be implicated in important house-keeping functions. The majority of these 19 families are uncharacterized or only with general function predicted. Interestingly enough, among those there are two genes that could be potentially involved in cell division of *Thermoproteales*, the only group of *Archaea* for which the cell division mechanism is not known yet [12]. One of these proteins is a ParA family ATPase (TTX_1301) involved in chromosome and plasmid partitioning [96] and the other is an actin-like protein (TTX_0752), the closest homolog of the major component of the cytoskeleton in *Eucarya* [97]. Another uncharacterized protein among these 19 is encoded in the same operon with actin, suggesting their functional relationships. These three proteins can be considered as prime candidates for a role in cell division of *Thermoproteales* (Table S7).

There are only six arCOGs that are present in *T. tenax* but not in other *Crenarchaeota*. One of them is DNA uptake protein DprA (TTX_0242), which is encoded in a predicted operon with another functionally related protein ComF (TTX_0243), which was never before detected in archaeal genomes and apparently has been transferred horizontally from *Bacteria*. Another example of potential horizontal gene transfer from *Bacteria* to *T. tenax* is the cytochrome b subunit of Ni,Fe-hydrogenase (Isp1, TTX_0032), which is also absent in other archaeal genomes.

We employed the tree representing the consensus view on archaeal taxonomy [98], [12], [99] and arCOG patterns (Figure S1) to reconstruct the gene repertoire of the common ancestor of the *T. tenax* and *Pyrobaculum* group and gene loss and gain events during the evolution of *T. tenax* lineage using the maximum-likelihood approach developed recently by Csurös and Miklos [100]. The estimated gene repertoire of the common ancestor of the *T. tenax* and *Pyrobaculum* group consists of 1,619 gene families. Proteins encoded in the *T. tenax* genome are assigned to 1,604 different arCOGs, whereas gene complement of the common ancestor of *Pyrobaculum* group is estimated as 1,768 families with the net gain of 149 genes. The estimated gene family drift for *T. tenax* is not very high, only 62 families were gained and 77 were lost, implying that the *T. tenax* genome shares 92% of gene families with the ancestor. For comparison, the *Pyrobaculum* group ancestor shares only 82% with the *Pyrobaculum*/*Thermoproteus* ancestor with further erosion of similarity within the *Pyrobaculum* clade. Similar estimates, restricted to metabolic genes, show that 34% *T. tenax* gene families are directly inherited from the ancestral metabolic repertoire, whereas *Pyrobaculum* group species only of 28% on average. The examples of functions (Table S7) include the exchange of genes with analogous functions, like the substitution of a number of amino acid ABC family transporters by PotE-like amino acid transporters (belonging to the amino acid/polyamine/organocation (APC) superfamily); and minor shifts in metabolic preferences, like acquisition of sugar transporters, which suggests an increasing role of sugar utilization for *T. tenax*. A few other gene families that are lost, have functional substitutes encoded in the genome. For example, the loss of ABC family phosphate transporter can be compensated by inorganic phosphate transporter. All the above indicates that *T. tenax* largely preserved the functional repertoire of the ancestor.

## Materials and Methods

### Strain and DNA preparation

*T. tenax* strain Kra 1 (DSM 2078^T^; NCBI Taxonomy ID 2271) cells were grown under autotrophic conditions as described previously [1]. Genomic DNA (gDNA) was prepared as described previously [101].

### Isolation of membranes and isolation of glycosylated proteins

Cells (400 ml) were spun down and membranes isolation and the purification of glycosylated membrane proteins were performed as described previously [79].

### Genome Sequencing

The genome was sequenced with a random (‘shotgun’) sequencing and assembly strategy [102] with gaps closed *via* primer walking on bridging plasmid clones, and by direct sequencing with chromosomal DNA as a template. Controlled fragmentation of the gDNA for cloning into plasmid vectors was done with a HydroShear (Gene Machines, San Carlos, CA). Fragments of 2.5 and 5 kbp average lengths, repectively, were cloned into TOPO subcloning vectors (Invitrogen, Carlsbad, CA) and sequenced from both ends after plasmid purification with QIAquick (Qiagen, Hilden, Germany) on Qiagen liquid handling stations according to the manufacturer̀s instructions. Sanger-type sequence reactions [103] were analyzed on ABI Prism 377 and 3700 systems (PE Biosystems) and processed for sequence quality (base calling) and assembly with the Phred/Phrap/Consed software package [104]–[106]. The final genome sequence was assembled from 17,638 sequence reads (including 1,244 primer walking sequences) with a mean trimmed read length of 616.2 nt, resulting in an 8-fold sequence coverage with an estimated error rate of less than 0.4×10^−4^.

### Sequence analysis and annotation

Analysis and sequence annotations have been performed as described previously (Table S8) [107]. GCskew analysis and localization of the putative start of the chromosomal replication was performed with the program GenSkew (http://mips.gsf.de/services/analysis/genskew). Repeats were identified and analyzed with REPuter [108]. High and low GC regions were identified by EMBOSS [109]. tRNA genes were located with tRNAscan-SE and GtRNAdb [91], [92]. ORFs were predicted by the expert program REGANOR [110], which is integrated into the GenDB package [111] and combines the gene finding programs GLIMMER [112] and CRITICA [113]. Curation and annotation of the genome were done with the help of the GENDB annotation package [111]. Curation by hand was performed in order to identify and remove false positive ORFs found by GLIMMER and CRITICA.

Annotation of the identified ORFs was accomplished on the basis of sequence similarity searches against a selection of sequence databases followed by manual expert curation. Similarity searches were performed by using blastx [114] against the NCBI nonredundant database on protein level [115], the Swissprot [116], KEGG [117], Clusters of Orthologous Groups (COG) [15] and the archaeal COG [16] database. Genes with a sufficient degree of similarity (cut-off 10E^−15^) were finally assigned to orthologous groups in COGs. ORFs shorter than 150 bp with best BLAST scores (E-values) higher than 10^−15^ were deleted from the final reported set of genes.

### Gene order and colinearity in *T. tenax* and *P. aerophilum* (indicated in Figure 3)

A quantitative co-linearity factor was calculated from the genomic positions (x and y coordinates) of each ortholog pair relative to O, the number of CDSs in the target genome, as follows: For each pair of neighbouring ORFs on the genome of *T. tenax* (xi, xi+1), the position of the corresponding orthologs on the genome of *P. aerophilum* (yi, yi+1) was used to calculate D = Min (|yi+1–yi|, O – |yi+1–yi|). The colinearity factor C is defined as C = ΣD/O.

### Comparative genomics and reconstruction of gene gain and loss events during the evolution of the *Thermoproteales* branch

Comparative genomic analysis of *T. tenax* proteome was done using the archaeal Clusters of Orthologous Groups database (arCOGs) [16], which was recently updated and contains 60 archaeal genomes (available at ftp://ftp.ncbi.nih.gov/pub/wolf/COGs/arCOG/) and the Integrated Microbial Genomes (img) suite at JGI [118]. Proteins of *T. tenax* were assigned to arCOGs using PSI-BLAST program [114] and arCOGs profiles. *T. tenax* representation in arCOGs was not included for delineation of archaeal (59 genomes, and 58 genomes with *N. equitans* excluded), crenarchaeal (18 genomes) and *Thermoproteales* (7 genomes) core arCOGs (families that are present in all genomes of the respective group).

Count software (http://www.iro.umontreal.ca/~csuros/gene_content/count.html) [100] was used to infer gene gain, loss and duplication rates on the branches of the species tree from the 59x8890 (*N. equitans* was excluded from consideration) matrix of phyletic patterns (containing a number of proteins in each genome assigned to a corresponding arCOG) by the likelihood maximization method based on a phylogenetic birth-and-death model. The tree representing the consensus view of archaeal phylogeny [98], [99], [16] was used as the guide topology (Figure S1). The model estimates probabilities for each arCOG to be present in each of the ancestral nodes and the rates of evolutionary events. For list of arCOGs present, lost or gained at the branches of interest, we used probability cutoff >0.5.

### Nucleotide sequence accession number

The genome sequence has been deposited in the EMBL Nucleotide Sequence Database under the accession number FN869859 and the MIGS compliant metadata in the Genomes Online Database (GOLD, www.genomesonline.org) [7] under the accession number GOLD Gc01285.

## Supporting Information

Figure S1 **Guide tree topology used for reconstruction of evolutionary events for the** ***Thermoproteales*** **lineage.** The tree represents the consensus view of archaeal phylogeny based on recent publications [16], [99], [98]. The *Thermoproteales* branch is shaded.
(PDF)

Figure S2 **Phylogeny of** ***Archaea*** **based on analysis of RNA polymerase subunits.** Maximum likelihood tree made from aligned sequences of the three largest RNA polymerase subunits: a, a′, and b as described previously [99]. Bootstrap support numbers are given at the nodes as a percentage (n = 10,000). Scale bars represent the average number of substitutions per residue.
(PDF)

Table S1 **Low G+C regions in the** ***T. tenax*** **genome.** Location in the genome, length and G+C content of the three identified regions are given.
(DOCX)

Table S2 **Clusters of regularly interspaced short palindromic repeats (CRISPR).**
(DOC)

Table S3 **Amino acid biosynthesis pathways.** The identified genes (sorted by respective biosynthesis pathways), their ID as well as gene name and annotation are given.
(DOCX)

Table S4 **TatFind (a) and FlaFind (b) positive ORFs in the** ***T. tenax*** **genome.** The tools TatFind (http://signalfind.org/tatfind.html) [75] and FlaFind (http://signalfind.org/flafind.html) [75] have been applied.
(DOCX)

Table S5 (**a**) Comparison of transporters in *Thermoproteus tenax*, *Pyrobaculum aerophilum* and *Sulfolobus solfataricus*. Classification according to the Paulsens transport database (http://www.membranetransport.org/) [76]. (**b**) *T. tenax* Substrate binding proteins.
(DOCX)

Table S6 (**a**) Annotated *T. tenax* tRNA genes. (**b**) Annotated genes encoding ribosomal proteins. Rps, ribosomal proteins. (**c**) Identified *T. tenax* tRNA synthetase genes.
(DOCX)

Table S7 **Comparative genomic analysis using arCOGs.** *Core genes*: The number of organisms in the corresponding archaeal lineages and number of proteins in individual genomes for respective arCOGs are given. *Gain-loss-expansion*: Results of the analysis of gene loss, gain and family expansion using arCOG data and COUNT software (http://www.iro.umontreal.ca/~csuros/gene_content/count.html) are given. Abbreviations: CREN - Crenarchaeota; EURY - Euryarchaeota; Tauma - Taumarchaeota; Korar - Korarchaeota; Nano - Nanoarchaeota; Thete - *Thermoproteus tenax*; All other organism abbreviations are explained in Figure S1.
(XLSX)

Table S8 **Annotated** ***T. tenax*** **genes.** The respective ORF ID, location and arCOG annotations as well as COG assignment are given. The last column provides GenBank GI accession numbers for the BLASTP best hits against NCBI Non-redundant database (e-value cutoff 0.001).
(XLSX)
