# Supplementary material for: The Complete Genome Sequence of Thermoproteus tenax: A Physiologically Versatile Member of the Crenarchaeota
Source: PLoS One. 2011 Oct 7;6(10):e24222. doi: 10.1371/journal.pone.0024222 (PMC3189178; doi:10.1371/journal.pone.0024222)
Supplement: Figure S1 — Guide tree topology used for reconstruction of evolutionary events for the Thermoproteales lineage. The tree represents the consensus view of archaeal phylogeny based on recent publications [16], [99], [98]. The Thermoproteales branch is shaded. (PDF) [file pone.0024222.s001.pdf]

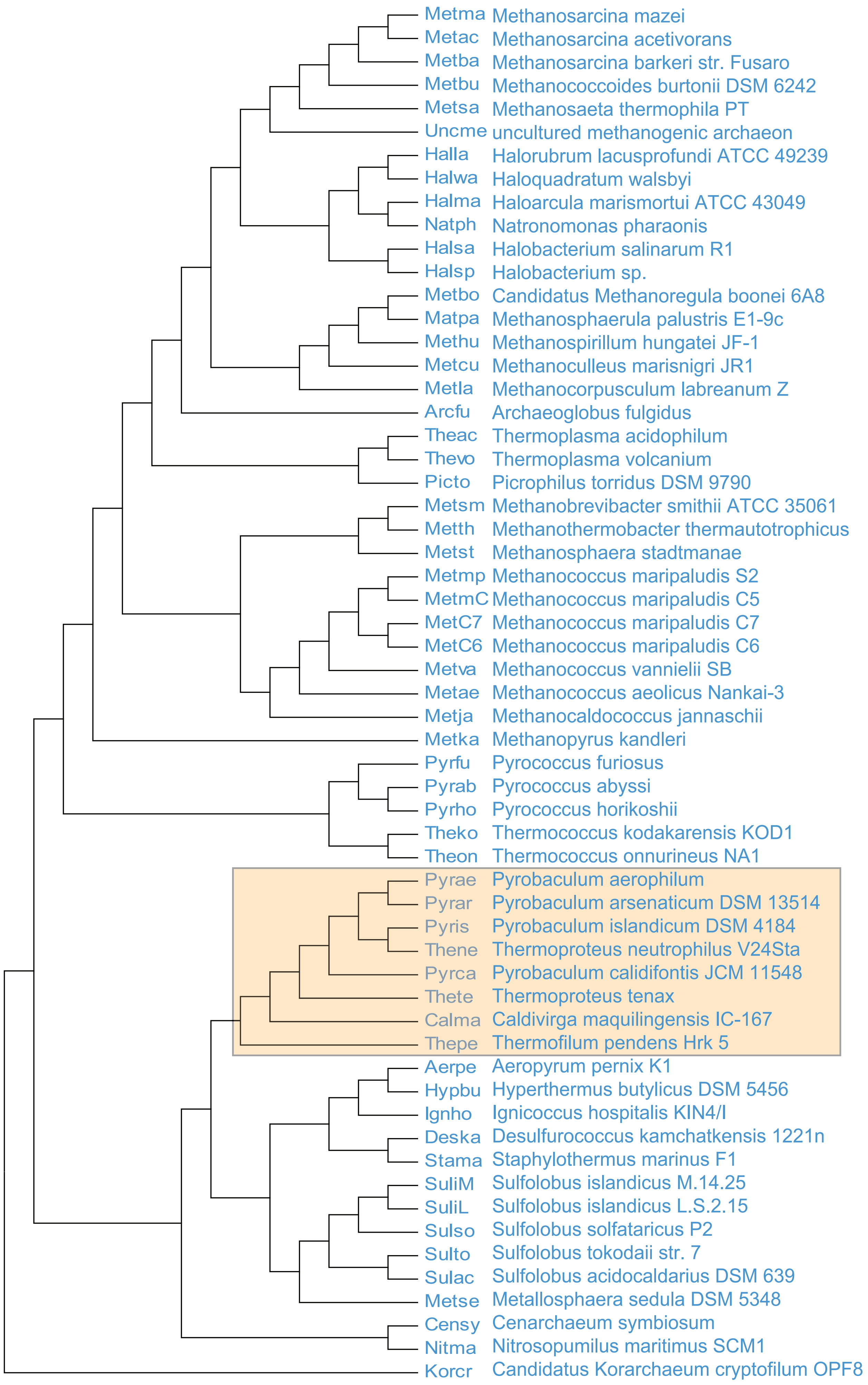

Metma Methanosarcina mazei
Metac Methanosarcina acetivorans
Metba Methanosarcina barkeri str. Fusaro
Metbu Methanococcoides burtonii DSM 6242
Metsa Methanosaeta thermophila PT
Uncme uncultured methanogenic archaeon
Halla Halorubrum lacusprofundi ATCC 49239
Halwa Haloquadratum walsbyi
Halma Haloarcula marismortui ATCC 43049
Natph Natronomonas pharaonis
Halsa Halobacterium salinarum R1
Halsp Halobacterium sp.
Metbo Candidatus Methanoregula boonei 6A8
Matpa Methanosphaerula palustris E1-9c
Methu Methanospirillum hungatei JF-1
Metcu Methanoculleus marisnigri JR1
Metla Methanocorpusculum labreanum Z
Arcfu Archaeoglobus fulgidus
Theac Thermoplasma acidophilum
Thevo Thermoplasma volcanium
Picto Picrophilus torridus DSM 9790
Metsm Methanobrevibacter smithii ATCC 35061
Metth Methanothermobacter thermautotrophicus
Metst Methanosphaera stadtmanae
Metmp Methanococcus maripaludis S2
MetmC Methanococcus maripaludis C5
MetC7 Methanococcus maripaludis C7
MetC6 Methanococcus maripaludis C6
Metva Methanococcus vannielii SB
Metae Methanococcus aeolicus Nankai-3
Metja Methanocaldococcus jannaschii
Metka Methanopyrus kandleri
Pyrfu Pyrococcus furiosus
Pyrab Pyrococcus abyssi
Pyrho Pyrococcus horikoshii
Theko Thermococcus kodakarensis KOD1
Theon Thermococcus onnurineus NA1
Pyrae Pyrobaculum aerophilum
Pyrar Pyrobaculum arsenaticum DSM 13514
Pyris Pyrobaculum islandicum DSM 4184
Thene Thermoproteus neutrophilus V24Sta
Pyrca Pyrobaculum calidifontis JCM 11548
Thete Thermoproteus tenax
Calma Caldivirga maquilingensis IC-167
Thepe Thermofilum pendens Hrk 5
Aerpe Aeropyrum pernix K1
Hypbu Hyperthermus butylicus DSM 5456
Ignho Ignicoccus hospitalis KIN4/I
Deska Desulfurococcus kamchatkensis 1221n
Stama Staphylothermus marinus F1
SuliM Sulfolobus islandicus M.14.25
SuliL Sulfolobus islandicus L.S.2.15
Sulso Sulfolobus solfataricus P2
Sulto Sulfolobus tokodaii str. 7
Sulac Sulfolobus acidocaldarius DSM 639
Metse Metallosphaera sedula DSM 5348
Censy Cenarchaeum symbiosum
Nitma Nitrosopumilus maritimus SCM1
Korcr Candidatus Korarchaeum cryptofilum OPF8

Figure S1. Guide tree topology used for reconstruction of evolutionary events for the *Thermoproteales* lineage. The tree represents the consensus view of archaeal phylogeny based on recent publications [16,99,98]. The *Thermoproteales* branch is shaded.
