# Supplementary material for: The Complete Genome Sequence of Thermoproteus tenax: A Physiologically Versatile Member of the Crenarchaeota
Source: PLoS One. 2011 Oct 7;6(10):e24222. doi: 10.1371/journal.pone.0024222 (PMC3189178; doi:10.1371/journal.pone.0024222)
Supplement: Figure S2 — Phylogeny of Archaea based on analysis of RNA polymerase subunits. Maximum likelihood tree made from aligned sequences of the three largest RNA polymerase subunits: a, a′, and b as described previously [99]. Bootstrap support numbers are given at the nodes as a percentage (n = 10,000). Scale bars represent the average number of substitutions per residue. (PDF) [file pone.0024222.s002.pdf]

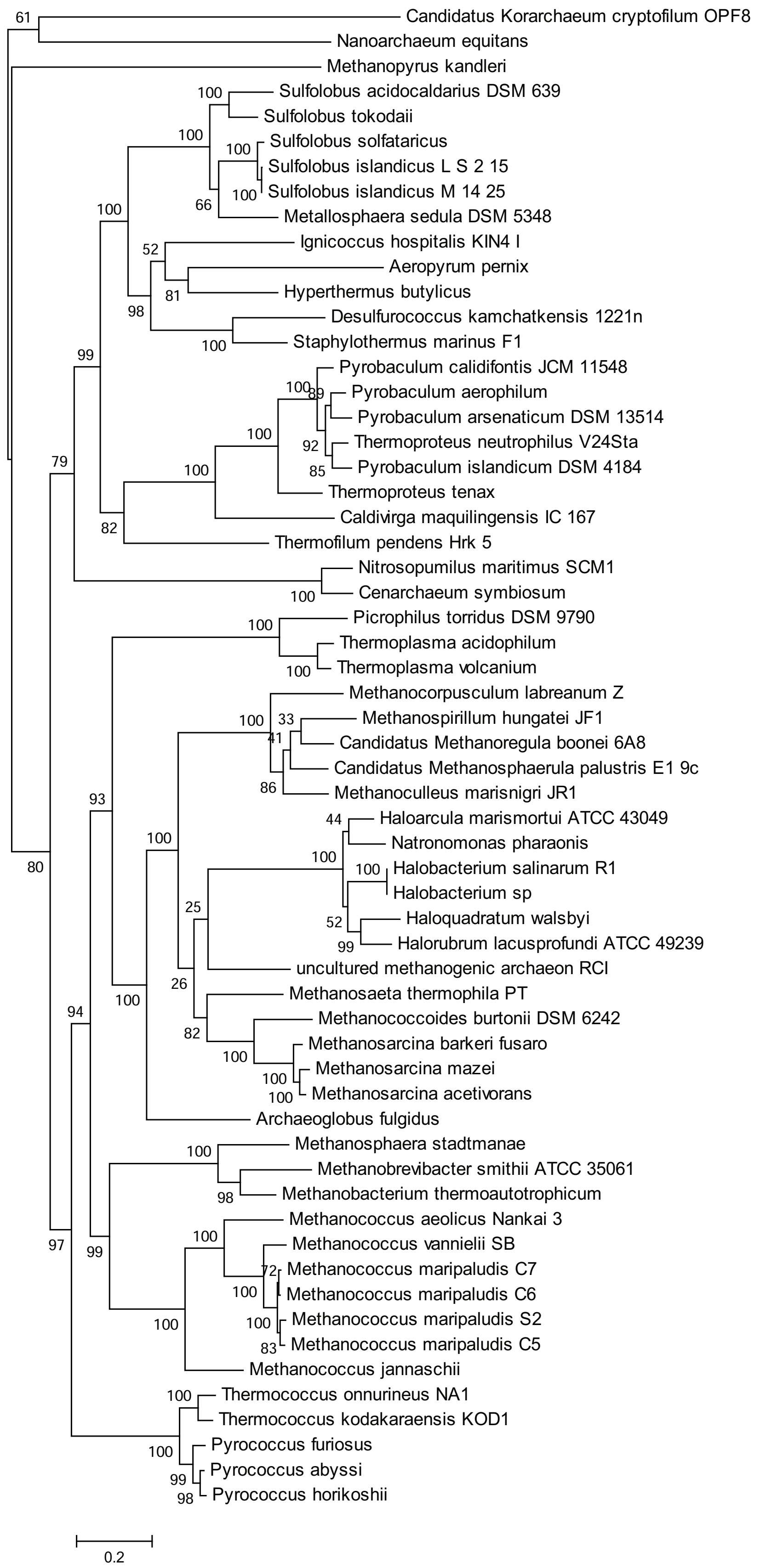
Candidatus Korarchaeum cryptofilum OPF8
Nanoarchaeum equitans
Methanopyrus kandleri
Sulfolobus acidocaldarius DSM 639
Sulfolobus tokodaii
Sulfolobus solfataricus
Sulfolobus islandicus L S 2 15
Sulfolobus islandicus M 14 25
Metallosphaera sedula DSM 5348
Ignicoccus hospitalis KIN4 I
Aeropyrum pernix
Hyperthermus butylicus
Desulfurococcus kamchatkensis 1221n
Staphylothermus marinus F1
Pyrobaculum calidifontis JCM 11548
Pyrobaculum aerophilum
Pyrobaculum arsenaticum DSM 13514
Thermoproteus neutrophilus V24Sta
Pyrobaculum islandicum DSM 4184
Thermoproteus tenax
Caldivirga maquilingensis IC 167
Thermofilum pendens Hrk 5
Nitrosopumilus maritimus SCM1
Cenarchaeum symbiosum
Picrophilus torridus DSM 9790
Thermoplasma acidophilum
Thermoplasma volcanium
Methanocorpusculum labreanum Z
Methanospirillum hungatei JF1
Candidatus Methanoregula boonei 6A8
Candidatus Methanosphaerula palustris E1 9c
Methanoculleus marisnigri JR1
Haloarcula marismortui ATCC 43049
Natronomonas pharaonis
Halobacterium salinarum R1
Halobacterium sp
Haloquadratum walsbyi
Halorubrum lacusprofundi ATCC 49239
uncultured methanogenic archaeon RCI
Methanosaeta thermophila PT
Methanococcoides burtonii DSM 6242
Methanosarcina barkeri fusaro
Methanosarcina mazei
Methanosarcina acetivorans
Archaeoglobus fulgidus
Methanosphaera stadtmanae
Methanobrevibacter smithii ATCC 35061
Methanobacterium thermoautotrophicum
Methanococcus aeolicus Nankai 3
Methanococcus vannielii SB
Methanococcus maripaludis C7
Methanococcus maripaludis C6
Methanococcus maripaludis S2
Methanococcus maripaludis C5
Methanococcus jannaschii
Thermococcus onnurineus NA1
Thermococcus kodakaraensis KOD1
Pyrococcus furiosus
Pyrococcus abyssi
Pyrococcus horikoshii
0.2

Figure S2. Phylogeny of *Archaea* based on analysis of RNA polymerase subunits. Maximum likelihood tree made from aligned sequences of the three largest RNA polymerase subunits: a, a’, and b as described previously [99]. Bootstrap support numbers are given at the nodes as a percentage (n = 10,000). Scale bars represent the average number of substitutions per residue.
