# Supplementary material for: The Complete Genome Sequence of Thermoproteus tenax: A Physiologically Versatile Member of the Crenarchaeota
Source: PLoS One. 2011 Oct 7;6(10):e24222. doi: 10.1371/journal.pone.0024222 (PMC3189178; doi:10.1371/journal.pone.0024222)
Supplement: Table S1 — Low G+C regions in the T. tenax genome. Location in the genome, length and G+C content of the three identified regions are given. (DOCX) [file pone.0024222.s003.docx]

**Table S1. Low G+C regions in the *T. tenax* genome.** Location in the genome, length and G+C content of the three identified regions are given.

| **Region** | **Position** | **Length [nt]** | **G+C [%]** | ***T. tenax* ORF IDs** |
| --- | --- | --- | --- | --- |
| 1 | 174.858 – 179.643 | 4786 | 38.3 | 0188 - 0190 |
| 2 | 519.631 – 524.276 | 4646 | 43.2 | 0583 - 0588 |
| 3 | 1.184.374 – 1.206.796 | 22423 | 46.8 | 1341 - 1363 |
