# Supplementary material for: The Complete Genome Sequence of Thermoproteus tenax: A Physiologically Versatile Member of the Crenarchaeota
Source: PLoS One. 2011 Oct 7;6(10):e24222. doi: 10.1371/journal.pone.0024222 (PMC3189178; doi:10.1371/journal.pone.0024222)
Supplement: Table S2 — Clusters of regularly interspaced short palindromic repeats (CRISPR). (DOC) [file pone.0024222.s004.doc]

**Table S2. Clusters of regularly interspaced short palindromic repeats (CRISPR).**

| Cluster | Repeat sequence*  [position in T. tenax genome] | Type | Direction | Repeat  [x-fold] | Size of spacers [bp] |
| --- | --- | --- | --- | --- | --- |
| 1 | GAATCTCAGATAGAGATTTGAAGG  [224,864-227,260] | I | reverse | 25 | 39-50 |
| 2 | AGTGGAAATCAAAAGATAGTAGAAAC  [316,783-317,791] | II | forward | 7 | 41-45 |
| 3 | GTGGAAATCAAAAGATAGTAGAAAG  [345,073-346,121] | II | reverse | 8 | 41-48 |
| 4 | GAATCTCAAAGAGAGGATTGAAAG  [1,075,638-1,078,562] | I | reverse | 34 | 37-57 |
| 5 | GAATCTCAAAGAGAGGATTGAAAG  [1,084,202-1,081,524] | I | forward | 32 | 38-51 |
| 6 | GAATCTCAAAAAGAGGATTGAAAG  [1,094,882-1,097,343] | I | reverse | 27 | 37-51 |
| 7 | GAATCTCAAAGAGAGGATTGAAAG  [1,102,723-1,104,458] | I | forward | 16 | 39-55 |

*Positions different from group consensus are underlined.
