# Supplementary material for: The Complete Genome Sequence of Thermoproteus tenax: A Physiologically Versatile Member of the Crenarchaeota
Source: PLoS One. 2011 Oct 7;6(10):e24222. doi: 10.1371/journal.pone.0024222 (PMC3189178; doi:10.1371/journal.pone.0024222)
Supplement: Table S3 — Amino acid biosynthesis pathways. The identified genes (sorted by respective biosynthesis pathways), their ID as well as gene name and annotation are given. (DOCX) [file pone.0024222.s005.docx]

**Table S3. Amino acid biosynthesis pathways.** The identified genes (sorted by respective biosynthesis pathways), their ID as well as gene name and annotation are given.

**ORF ID Gene Enzyme EC No.**

*Alanine*

from pyruvate and glutamate

TTX_0923 *msat* putative multiple substrate aminotransferase 2.6.1.1

*Arginine*

from carbamoyl-phosphate or aspartic acid

TTX_0468 *carA* carbamoyl-phosphate synthase, small-subunit 6.3.5.5

TTX_0040 *carB* carbamoyl-phosphate synthase, large-subunit 6.3.5.5

TTX_0807 *arcC* carbamate kinase 2.7.2.2

TTX_0091 *argF* ornithine carbamoyltransferase 2.1.3.3

TTX_0123 *argG* argininosuccinate synthase 6.3.4.5

TTX_0467 *argH* argininosuccinate lyase 4.3.2.1

*Aromatic amino acids (Tryptophan, tyrosine and phenylalanine)*

Shikimate synthesis

TTX_1756 *aroG* phospho-2-dehydro-3-deoxyheptonate aldolase 2.5.1.54

TTX_1752 *aroB* 3-dehydroquinate synthase 4.2.3.4

TTX_1749 *aroD* 3-dehydroquinate dehydratase 4.2.1.10

TTX_1748 *aroE* shikimate 5-dehydrogenase 1.1.1.25

Chorismate synthesis

TTX_1746 *aroK* shikimate kinase 2.7.1.71

TTX_1751 *aroA* 3-phosphoshikimate 1-carboxyvinyltransferase 2.5.1.1

TTX_1747 *aroC* chorismate synthase 4.2.3.5

Antranilate route (Tryptophan)

TTX_1842 *trpG* anthranilate synthase, component II 4.1.3.27

TTX_1843 *trpE* anthranilate synthase, component I 4.1.3.27

TTX_1404 *trpD* anthranilate phosphoribosyltransferase 2.4.2.18

TTX_1405 phosphoribosyl anthranilate isomerase 5.3.1.24

TTX_1841 *trpC* indole-3-glycerol phosphate synthase 4.1.1.48

TTX_1402 *trpA* tryptophan synthase, alpha-subunit 4.2.1.20

TTX_1403 *trpB* tryptophan synthase, beta-subunit 4.2.1.20

TTX_0296 *trpB* tryptophan synthase, beta-subunit 4.2.1.20

Prephenate route (Tyrosine, phenylalanine)

TTX_0528 *pheA* chorismate mutase / prephenate dehydratase 5.4.99.5/

(bifunctional enzyme) 4.2.1.51

TTX_1729 prephenate dehydrogenase 1.3.1.12

TTX_1733 *aspC* aspartate aminotransferase 2.6.1.1

TTX_1750 *aspB-2* aspartate aminotransferase 2.6.1.1

*Asparagine*

from aspartic acid

TTX_0300 *asnB* putative asparagine synthetase 6.3.5.4

*Aspartic acid*

from oxalacetate

TTX_1733 *aspC* aspartate aminotransferase

TTX_1750 *aspB-2* aspartate aminotransferase

TTX_0849 *aspC* aspartate aminotransferase 2.6.1.1

TTX_0923 *msat* multiple substrate aminotransferase 2.6.1.1

*Branched chain amino acids* (valine, leucine and isoleucine)

Leucine and valine from pyruvate

TTX_0130 *ilvB* acetolactate synthase, large-subunit 2.2.1.6

TTX_0131 *ilvN* acetolactate synthase, small-subunit 2.2.1.6

TTX_0132 *ilvC* ketol-acid reductoisomerase 1.1.1.86

TTX_0789 *ilvD* dihydroxy-acid dehydratase 4.2.1.9

TTX_0923 multiple substrate aminotransferase

TTX_0133 *ilvE* branched-chain amino acid aminotransferase 2.6.1.42

TTX_0128 *leuA-1* 2-isopropylmalate synthase 1 2.3.3.13

TTX_0134 *leuA-2* 2-isopropylmalate synthase 2 2.3.3.13

TTX_0202 *leuA-3* 2-isopropylmalate synthase 3 2.3.3.13

TTX_0129 *leuD* 3-isopropylmalate dehydratase, small-subunit 4.2.1.33

TTX_0136 *leuC* 3-isopropylmalate dehydratase, large-subunit 4.2.1.33

TTX_0126 *leuB* 3-isopropylmalate dehydrogenase 1.1.1.85

TTX_0133 *ilvE* branched-chain amino acid aminotransferase 2.6.1.42

Isoleucine from threonine

TTX_0924 *ilvA* threonine dehydratase 4.3.1.19

TTX_0130 *ilvB* acetolactate synthase large-subunit 2.2.1.6

TTX_0131 *ilvN* acetolactate synthase, small-subunit 2.2.1.6

TTX_0132 *ilvC* ketol-acid reductoisomerase 1.1.1.86

TTX_0789 *ilvD* dihydroxy-acid dehydratase 4.2.1.9

TTX_0133 *ilvE* branched-chain amino acid aminotransferase 2.6.1.42

*Cysteine*

TTX_0191 *cysM* cysteine synthase 2.5.1.47

*Glutamic acid*

from α-ketoglutarate

TTX_1095 *gdhA* glutamate dehydrogenase 1.4.1.3

TTX_1127 *gdhA* glutamate dehydrogenase 1.4.1.3

TTX_0628 *gltB2* putative glutamate synthase domain 2 1.4.1.13

TTX_0629 *gltB1/3* putative glutamate synthase domain 1+3 1.4.1.13

TTX_1987 *gltD* glutamate synthase small subunit 1.4.1.13

*Glutamine*

from glutamic acid

TTX_0627 *glnA* glutamine synthetase 6.3.1.2

*Glycine*

from 3-phosphoglycerate

TTX_2093 *serA* D-3-phosphoglycerate dehydrogenase 1.1.1.95

TTX_2092 putative aminotransferase 2.6.1.-

TTX_0682 *serB* phosphoserine phosphatase 3.1.3.3

*Histidine*

TTX_1005 *prsA* ribose-phosphate pyrophosphokinase 2.7.6.1

TTX_0779 *hisG* ATP phosphoribosyltransferase 2.4.2.17

TTX_0372 ATPase

TTX_0781a *hisI*2 phosphoribosyl-ATP-pyrophosphatase 3.6.1.31

TTX_0781 *hisI*1 phosphoribosyl-AMP cyclohydrolase 3.5.4.19

TTX_0785 *hisA* phosphoribosylformimino-5-aminoimidazole

carboxamide ribotide isomerase 5.3.1.1

TTX_0778 *hisH* imidazole glycerol phosphate synthase, 2.4.2.-

glutamine amidotransferase subunit H

TTX_0783 *hisF* imidazole glycerol phosphate synthase, cyclase

subunit F 4.1.3.-

TTX_0784 *hisB* imidazoleglycerol-phosphate dehydratase 4.2.1.19

TTX_0780 *hisC* histidinol-phosphate aminotransferase 2.6.1.9

TTX_1708 phosphohydrolase, HD superfamily

TTX_0782 *hisD* histidinol dehydrogenase 1.1.1.23

*Lysine*

TTX_0122 *lysW* lysine biosynthesis regulator protein (LysW)

TTX_0466 *lysX / rimK* lysine biosynthesis protein (LysX) /

ribosomal protein S6 modification

TTX_0121 *lysZ / argB* acetylaminoadipate / acetylglutamate kinase 2.7.2.- /

2.7.2.8

TTX_0120 argC / lysY N-acetyl-gamma- aminoadipyl-phosphate / 1.2.1.- /

N-acetyl-gamma-glutamyl-phosphate reductase 1.2.1.38

TTX_0623 *lysJ / argD* acetyl-lysine / acetylornithine aminotransferase 2.6.1.- /

2.6.1.11

TTX_0118 lysK acetyl-lysine deacetylase 3.5.1.- /

3.5.1.16

*Methionine*

from homocysteine

TTX_1021 *metE* methionine synthase 2.1.1.14

(homocysteine methyltransferase)

Homocysteine from cysteine

TTX_2096 *cth* cysthatione gamma lyase 4.4.1.1

*Proline*

from glutamic acid

TTX_1787 *aldh-1 / putA* Aldehyde dehydrogenase / 1-pyrroline-5-carboxylate 1.2.1.- /

dehydrogenase 1.5.1.12

TTX_1730 *proC* pyrroline-5-carboxylate reductase 1.5.1.2

from ornithine

TTX_2070 *ocd*  ornithine cyclodeaminase 4.3.1.12

TTX_0618 *ocd*  ornithine cyclodeaminase 4.3.1.12

*Serine*

from glycine

TTX_1905 *glyA* serine hydroxymethyltransferase 2.1.2.1

*Threonine*

from aspartate

TTX_2010 *lysC* aspartate kinase 2.7.2.4

TTX_2009 *asd* aspartyl-β-semialdehyde dehydrogenase 1.2.1.11

TTX_2007 *hom* homoserine dehydrogenase 1.1.1.3

TTX_0477 *thrB* homoserine kinase 2.7.1.39

TTX_2011 *thrC* threonine synthase 4.2.3.1

TTX_0141 *thrC-1* putative threonine synthase 4.2.3.1

TTX_0361 *thrC-2* putative threonine synthase 4.2.3.1
