# Supplementary material for: The Complete Genome Sequence of Thermoproteus tenax: A Physiologically Versatile Member of the Crenarchaeota
Source: PLoS One. 2011 Oct 7;6(10):e24222. doi: 10.1371/journal.pone.0024222 (PMC3189178; doi:10.1371/journal.pone.0024222)
Supplement: Table S4 — TatFind (a) and FlaFind (b) positive ORFs in the T. tenax genome. The tools TatFind (http://signalfind.org/tatfind.html) [75] and FlaFind (http://signalfind.org/flafind.html) [75] have been applied. (DOCX) [file pone.0024222.s006.docx]

**Table S4.a and S4.b. TatFind (a) and FlaFind (b) positive ORFs in the *T. tenax* genome.** The tools TatFind (<http://signalfind.org/tatfind.html>) [72] and FlaFind (http://signalfind.org/flafind.html) [75] have been applied.

**Table S4.a.**

| **ORF ID** | **Gene** | **Proposed function** |
| --- | --- | --- |
| TTX_0031 | hynS | Ni,Fe-hydrogenase I small subunit |
| TTX_0046 | sreA1 | sulfur (polysulfide) reductase, large subunit A, Mo-FeS protein |
| TTX_0091 | argF | ornithine carbamoyltransferase |
| TTX_0319 | soxL | Rieske iron-sulfur protein |
| TTX_0365 |  | hypothetical protein |
| TTX_0393 | livK | ABC-type branched-chain amino acid transport system, periplasmic component |
| TTX_1203 | fdhA | formate dehydrogenase alpha subunit |

**Table S4.b**

| **ORF ID** | **Gene** | **Proposed function** |
| --- | --- | --- |
| TTX_0113 | arsA2 | anion (arsenite)-transporting ATPase |
| TTX_0295 |  | hypothetical protein |
| TTX_0321 | cbsA | cytochrome b558/566, subunit A |
| TTX_0332 |  | hypothetical protein |
| TTX_0451 |  | Uncharacterized conserved membrane protein |
| TTX_0498 |  | hypothetical protein |
| TTX_0723 |  | conserved hypothetical protein |
| TTX_0876 |  | fragment of inactivated transposase |
| TTX_0966 |  | predicted membrane component of Type II/IV secretion |
| TTX_0967 |  | pilin-like component of Type II/IV secretion system, PilA family |
| TTX_0970 |  | predicted component of Type II/IV secretion system |
| TTX_1130 |  | Type II/IV secretion system component, FlgK family |
| TTX_1306 |  | hypothetical protein |
| TTX_1846 |  | conserved hypothetical protein |
| TTX_2041 |  | conserved hypothetical protein |
