# Supplementary material for: The Complete Genome Sequence of Thermoproteus tenax: A Physiologically Versatile Member of the Crenarchaeota
Source: PLoS One. 2011 Oct 7;6(10):e24222. doi: 10.1371/journal.pone.0024222 (PMC3189178; doi:10.1371/journal.pone.0024222)
Supplement: Table S5 — (a) Comparison of transporters in Thermoproteus tenax, Pyrobaculum aerophilum and Sulfolobus solfataricus. Classification according to the Paulsens transport database (http://www.membranetransport.org/) [76]. (b) T. tenax Substrate binding proteins. (DOCX) [file pone.0024222.s007.docx]

**Table S5.a.** **Comparison of transporters in *Thermoproteus tenax*, *Pyrobaculum aerophilum* and *Sulfolobus solfataricus.*** Classification according to the Paulsens transport database, <http://www.membranetransport.org/>) [76].

|  | ***T. tenax*** | ***P. aerophilum*** | ***S. solfataricus*** |
| --- | --- | --- | --- |
| Genome Size (Mb) | 1.84 | 2.22 | 2.99 |
| Total numbers of transport proteins | 133 | 146 | 191 |
| No of transporters/Mb genome | 73.9 | 65.77 | 63.88 |
| **ATP dependent** | 26 (19.5%)^+^ | 47 (32.2%) | 40 (20.9%)^+^ |
| ABC family* | 23 | 42 | 37 |
| ArsAB family | 3 | 2 | - |
| **Ion channels** | 4(3%) | 4 (2.7%) | 7 (3.7%) |
| CIC family | 1 |  | 1 |
| MscC family | 3 | 4 | 4 |
| **Secondary transporters** | 53(39.9%) | 25 (17.1%) | 81 (42.4%) |
| Amt family | 2 | - | 1 |
| APC family  (Amino acid transporters) | 6 | 1 | 14 |
| CaCA family | 1 | 1 | 1 |
| CDF family | 1 | 3 | 2 |
| CPA2 family | 1 | 1 | 3 |
| DMT family | 5 | 3 | 2 |
| LctP family | 1 | - | 1 |
| MFS family | 25 | 12 | 45 |
| NCS2 family | 1 | - | - |
| MIT family | 1 | - | - |
| NiCoT family | 1 | - | 1 |
| Nramp family | 1 | - | 2 |
| RhtB family | 1 | - | - |
| RND family | 1 | 1 | 1 |
| SSS family | 1 | - | 2 |
| Uncharacterized | 4 | 1 | - |

^+^Families, which are either present in *S. solfataricus* or *P. aerophilum* are not shown in the table.

*Transport proteins functioning as group are only counted once.

**Table S5.b. Substrate binding proteins.**

|  | **Predicted substrate** | **Location of**  **ST-linker** | **Predicted TMDs** | **Identified by MS** |
| --- | --- | --- | --- | --- |
| TTX_0054 | Sugar | N-terminal | 1 |  |
| TTX_0083 | Dipeptides | N-terminal | 1 | yes |
| TTX_0155 | Oligopeptides | C-terminal | 2 | yes |
| TTX_0188 | Sugar | N-terminal | 1 |  |
| TTX_0258 | Iron | N-terminal | 1 |  |
| TTX_0393 |  | N-terminal | 1 |  |
| TTX_0482 | Branched amino acids | N-terminal | 1 | yes |
| TTX_0490 | Manganese | C-terminal (QT) | 2 |  |
| TTX_0853 | Sulfate | N-terminal | 1 |  |
| TTX_1120 | Sugar | N-terminal (QT) | 1 |  |
| TTX_1140 | Branched amino acids | N-terminal | 1 | yes |
| TTX_1143 | Branched amino acids | N-terminal | 1 |  |
| TTX_1780 | Sugar | N-terminal | 1 |  |
| TTX_1992 | Iron | N-terminal | 1 |  |
