# Supplementary material for: The Complete Genome Sequence of Thermoproteus tenax: A Physiologically Versatile Member of the Crenarchaeota
Source: PLoS One. 2011 Oct 7;6(10):e24222. doi: 10.1371/journal.pone.0024222 (PMC3189178; doi:10.1371/journal.pone.0024222)
Supplement: Table S6 — (a) Annotated T. tenax tRNA genes. (b) Annotated genes encoding ribosomal proteins. Rps, ribosomal proteins. (c) Identified T. tenax tRNA synthetase genes. (DOCX) [file pone.0024222.s008.docx]

**Table S6.a Annotated *T. tenax* tRNA genes.**

| **tRNA-ID** | **Position** |  | **Type** | **Anticodon** | **Intron position*** |  |
| --- | --- | --- | --- | --- | --- | --- |
|  | begin | end |  |  | begin | end |
| TTX_trna_1 | 16066 | 16173 | Met | CAT |  |  |
| TTX_trna_2 | 144008 | 144079 | Ala | GGC |  |  |
| TTX_trna_3 | 240023 | 240094 | Pseudo | GCG |  |  |
| TTX_trna_4 | 495405 | 495489 | Ser | GCT |  |  |
| TTX_trna_5 | 730801 | 730877 | Trp | CCA |  |  |
| TTX_trna_6 | 736409 | 736500 | Glu | TTC |  |  |
| TTX_trna_7 | 820042 | 820138 | Ile | GAT |  |  |
| TTX_trna_8 | 876950 | 877079 | Glu | CTC |  |  |
| TTX_trna_9 | 919908 | 919991 | Ser | TGA |  |  |
| TTX_trna_10 | 1252042 | 1252156 | Tyr | GTA |  |  |
| TTX_trna_11 | 1289234 | 1289341 | Gln | TTG | 1289290 | 1289306 |
| TTX_trna_12 | 1294478 | 1294571 | Met | CAT |  |  |
| TTX_trna_13 | 1374638 | 1374725 | Ala | TGC |  |  |
| TTX_trna_14 | 1423104 | 1423187 | Ser | GGA |  |  |
| TTX_trna_15 | 1494415 | 1494516 | Ser | CGA | 1494453 | 1494470 |
| TTX_trna_16 | 1503893 | 1503965 | Val | GAC |  |  |
| TTX_trna_17 | 1505997 | 1506095 | Gly | CCC |  |  |
| TTX_trna_18 | 1510962 | 1511063 | Leu | TAG |  |  |
| TTX_trna_19 | 1565939 | 1566030 | Thr | GGT |  |  |
| TTX_trna_20 | 1685631 | 1685740 | Thr | TGT |  |  |
| TTX_trna_21 | 1729009 | 1729129 | Gly | TCC |  |  |
| TTX_trna_22 | 1771869 | 1771798 | His | GTG |  |  |
| TTX_trna_23 | 1728670 | 1728551 | Lys | CTT |  |  |
| TTX_trna_24 | 1728492 | 1728399 | Arg | TCT | 1728453 | 1728435 |
| TTX_trna_25 | 1626841 | 1626730 | Pro | GGG | 1626783 | 1626766 |
| TTX_trna_26 | 1621993 | 1621901 | Arg | GCG | 1621953 | 1621937 |
| TTX_trna_27 | 1591222 | 1591110 | Thr | CGT | 1591162 | 1591146 |
| TTX_trna_28 | 1560706 | 1560585 | Pro | CGG |  |  |
| TTX_trna_29 | 1499962 | 1499860 | Leu | CAG |  |  |
| TTX_trna_30 | 1497453 | 1497351 | Leu | GAG |  |  |
| TTX_trna_31 | 1423334 | 1423250 | Leu | CAA |  |  |
| TTX_trna_32 | 1403947 | 1403855 | Pro | TGG |  |  |
| TTX_trna_33 | 1383958 | 1383839 | Lys | TTT | 1383896 | 1383875 |
| TTX_trna_34 | 1381178 | 1381058 | Arg | CCT | 1381115 | 1381094 |
| TTX_trna_35 | 1294391 | 1294304 | Ala | CGC |  |  |
| TTX_trna_36 | 1215120 | 1215016 | Asn | GTT |  |  |
| TTX_trna_37 | 1213773 | 1213683 | Gln | CTG |  |  |
| TTX_trna_38 | 876533 | 876422 | Asp | GTC |  |  |
| TTX_trna_39 | 766864 | 766762 | Arg | CCG |  |  |
| TTX_trna_40 | 725282 | 725182 | Val | TAC | 725245 | 725218 |
| TTX_trna_41 | 603212 | 603122 | Cys | GCA |  |  |
| TTX_trna_42 | 289669 | 289598 | Phe | GAA |  |  |
| TTX_trna_43 | 281175 | 281091 | Leu | TAA |  |  |
| TTX_trna_44 | 60207 | 60135 | Val | CAC |  |  |
| TTX_trna_45 | 60067 | 59973 | Met | CAT |  |  |
| TTX_trna_46 | 40937 | 40818 | Gly | GCC | 40897 | 40878 |
| TTX_trna_47 | 39371 | 39280 | Arg | TCG | 39333 | 39316 |

*Only introns located between position 37 and 38 are listed.

**Table S6.b. Annotated genes encoding ribosomal proteins.** Rps, ribosomal proteins.

|  | **Rps** | **ORF ID** | **Rps** | **ORF ID** |
| --- | --- | --- | --- | --- |
| **Small subunit** | S2p | TTX_1898 | S3ae | TTX_0716 |
|  | S3p | TTX_1554 | S4e | TTX_2098 |
|  | S4p | TTX_1563 | S6e | TTX_0099 |
|  | S5p | TTX_1410 | S8e | TTX_2084 |
|  | S7p | TTX_1852 | S17e | TTX_1925 |
|  | S8p | TTX_1817 | S19e | TTX_0582 |
|  | S9p | TTX_1669 | S24e | TTX_0913 |
|  | S10p | TTX_0012 | S25e | TTX_0177 |
|  | S11p | TTX_1650 | S26e | TTX_0164 |
|  | S12p | TTX_1673 | S27ae | TTX_0912 |
|  | S13p | TTX_1562 | S27e | TTX_0576 |
|  | S14p | TTX_1816 | S28e | TTX_2001 |
|  | S15p | TTX_0719 | S30e | TTX_0151/TTX_0161 |
|  | S17p | TTX_1530 |  |  |
|  | S19p | TTX_1529 |  |  |
| **Large subunit** | L1p | TTX_1805 | L7ae | TTX_1999 |
|  | L2p | TTX_1894 | L10e | TTX_0511 |
|  | L3p | TTX_1742 | L13e | TTX_1480 |
|  | L4p | TTX_1741 | L14e | TTX_1857 |
|  | L5p | TTX0298a | L15e | TTX_0936 |
|  | L6p | TTX_1424 | L18e | TTX_1671 |
|  | L10p | TTX_1804 | L19e | TTX_1825 |
|  | L11p | TTX_1806 | L21e | TTX_2081 |
|  | L12p | TTX_1506 | L24e | TTX_2000 |
|  | L13p | TTX_1670 | L30e | TTX_1675 |
|  | L14p | TTX_1628 | L31e | TTX_0585 |
|  | L15p | TTX_1415 | L32e | TTX_1822 |
|  | L18p | TTX_1826 | L34e | TTX_1989 |
|  | L22p | TTX_1500 | L37ae | TTX_0822 |
|  | L23p | TTX_1740 | L37e | TTX_1917a |
|  | L24p | TTX_0915 | L39e | TTX_0584a |
|  | L29p | TTX_1553 | L40e | TTX_2018 |
|  | L30p | TTX_1409 | L44e | TTX_0575 |
|  |  |  | LXa | TTX_1811 |

**Table S6.c. Identified *T. tenax* tRNA synthetase genes.**

| **ORF ID** | **gene** | **tRNA synthetase** |
| --- | --- | --- |
| TTX_0163 | *proS* | prolyl-tRNA synthetase |
| TTX_0219 | *trpS* | tryptophanyl-tRNA synthetase |
| TTX_0464 | *pheS* | phenylalanyl-tRNA synthetase alpha chain |
| TTX_1527 | *pheT* | phenylalanyl-tRNA synthetase beta chain |
| TTX_0514 | *alaS* | alanyl-tRNA synthetase |
| TTX_0655 | *metS* | methionyl-tRNA synthetase |
| TTX_0657 | *metS* | methionyl-tRNA synthetase |
| TTX_0808 | *hisS* | histidyl-tRNA synthetase |
| TTX_0858 | *ileS* | isoleucyl-tRNA synthetase |
| TTX_0930 | *valS* | valyl-tRNA synthetase |
| TTX_0953 | *aspS* | aspartyl-tRNA synthetase |
| TTX_0996 &_0997 | *gltX* | glutamyl- and glutaminyl-tRNA synthetase |
| TTX_1000 | *asnS* | asparaginyl-tRNA synthetase |
| TTX_1058 | *leuS* | leucyl-tRNA synthetase |
| TTX_1538 | *thrS* | threonyl-tRNA synthetase |
| TTX_1596 | *glyS* | glycyl-tRNA synthetase (class II) |
| TTX_1618 | *tyrS* | tyrosyl-tRNA synthetase |
| TTX_1640 | *serS* | seryl-tRNA synthetase |
| TTX_1755 | *tyrS* | tyrosyl-tRNA synthetase |
| TTX_1890 | *cysS* | cysteinyl-tRNA synthetase |
| TTX_1998 | *argS* | arginyl-tRNA synthetase |
| TTX_2056 | *lysS* | lysyl-tRNA synthetase (class II) |
